# The TaSnRK1‐TabHLH489 module integrates brassinosteroid and sugar signalling to regulate the grain length in bread wheat

**DOI:** 10.1111/pbi.14319

**Published:** 2024-02-27

**Authors:** Jinyang Lyu, Dongzhi Wang, Na Sun, Fan Yang, Xuepeng Li, Junyi Mu, Runxiang Zhou, Guolan Zheng, Xin Yang, Chenxuan Zhang, Chao Han, Guang‐Min Xia, Genying Li, Min Fan, Jun Xiao, Ming‐Yi Bai

**Affiliations:** ^1^ The Key Laboratory of Plant Development and Environmental Adaptation Biology, Ministry of Education, School of Life Sciences Shandong University Qingdao China; ^2^ State Key Laboratory of Plant Cell and Chromosome Engineering, Institute of Genetics and Developmental Biology Chinese Academy of Sciences Beijing China; ^3^ Crop Research Institute Shandong Academy of Agricultural Sciences Jinan China; ^4^ University of Chinese Academy of Sciences Beijing China; ^5^ Centre of Excellence for Plant and Microbial Science (CEPAMS) JIC‐CAS Beijing China

**Keywords:** wheat grain length, brassinosteroid, sugar, TabHLH489, SnRK1

## Abstract

Regulation of grain size is a crucial strategy for improving the crop yield and is also a fundamental aspect of developmental biology. However, the underlying molecular mechanisms governing grain development in wheat remain largely unknown. In this study, we identified a wheat atypical basic helix–loop–helix (bHLH) transcription factor, TabHLH489, which is tightly associated with grain length through genome‐wide association study and map‐based cloning. Knockout of *TabHLH489* and its homologous genes resulted in increased grain length and weight, whereas the overexpression led to decreased grain length and weight. TaSnRK1α1, the α‐catalytic subunit of plant energy sensor SnRK1, interacted with and phosphorylated TabHLH489 to induce its degradation, thereby promoting wheat grain development. Sugar treatment induced TaSnRK1α1 protein accumulation while reducing TabHLH489 protein levels. Moreover, brassinosteroid (BR) promotes grain development by decreasing *TabHLH489* expression through the transcription factor BRASSINAZOLE RESISTANT1 (BZR1). Importantly, natural variations in the promoter region of *TabHLH489* affect the TaBZR1 binding ability, thereby influencing *TabHLH489* expression. Taken together, our findings reveal that the TaSnRK1α1‐TabHLH489 regulatory module integrates BR and sugar signalling to regulate grain length, presenting potential targets for enhancing grain size in wheat.

## Introduction

Bread wheat (*Triticum aestivum* L.) is a vital staple food crops worldwide, providing sustenance for 40% of the global population (Gupta *et al*., 2008). With the demand for food increasing and farmland decreasing, breeding for increased wheat yield has become a crucial objective. Among the various components, grain weight is one of the intensively studied traits due to its higher stability and heritability even under changing environmental conditions. Grain weight is determined by grain size and grain‐filling rate, with genetic pathways such as proteasomal degradation, G‐protein signalling and phytohormone signalling mainly associated with controlling grain size (Li and Yang, 2017). These pathways are genetically conserved in monocotyledonous and dicotyledonous plant species despite some differences in functional mode. In regulating grain size, the components of the ubiquitination‐proteasome pathway play critical roles, such as GW2 (Hao *et al*., 2021; Song *et al*., 2007), WTG1/OsOTUB1 (Huang *et al*., 2017; Wang *et al*., 2017), OsUBP15 (Shi *et al*., 2019) and HDR3 (Gao *et al*., 2021). All subunits of heterotrimeric G proteins complex are involved in regulating grain size in rice (Cui *et al*., 2020; Liu *et al*., 2022; Sun *et al*., 2018), including Gα subunit (D1/RGA1), Gβ subunit (RGB1) and Gγ subunits (RGG1, RGG2, GS3, DEP1 and OsGGC2).

The plant steroid phytohormone brassinosteroid (BR) has been reported to play critical roles in grain development (Tong and Chu, 2018; Wu *et al*., 2008). BR binding to the extracellular domain of the plasma membrane localized receptor kinase BRASSINOSTEROID INSENSITIVE1 (BRI1) triggers a series of phosphorylation and dephosphorylation to inhibit the activity of GSK3‐like kinase BRASSINOSTEROID‐INSENSITIVE2 (BIN2) and then activating the BRASSINAZOLE RESISTANT1 (BZR1) family transcription factors, which directly regulate BR‐responsive gene expression and plant development (Kim and Wang, 2010; Sun *et al*., 2010; Yu *et al*., 2011). The rice defective BR biosynthesis or signalling mutants displayed the reduced grain length and decreased grain weight, such as *brd1, brd2, d2, d61, gw5, gsk2, gl2* and *Osbzr1* (Bai *et al*., 2007; Che *et al*., 2015; Hong *et al*., 2002, 2003; Huang *et al*., 2022; Liu *et al*., 2017; Yamamuro *et al*., 2000). Similar, genes affecting BR biosynthesis and signalling in wheat have also been shown to control grain size. For example, the wheat BR‐deficient *Tad11* mutant produced shorter grain compared to wild type and knock‐down the expression of *TaBRI1* or increasing *TaGSK3* protein stability in *Tasg‐D1* mutant displayed the less sensitivity to exogenous BR and decreased grain size (Cheng *et al*., 2020; Xu *et al*., 2022).

Sucrose nonfermenting‐1‐related protein kinase 1 (SnRK1) is an evolutionarily conserved energy sensor in plants that shares homology with SNF1 in yeasts and AMP‐activated protein kinase (AMPK) in mammals. SnRK1 kinase functions with a heterotrimeric complex, containing catalytic α subunit, regulatory β subunit and γ subunit. SnRK1 promotes catabolism and reduces anabolism to maintain cellular energy homeostasis under stress conditions by phosphorylating key enzymes of diverse metabolic processes and master regulators of various signalling pathways. Additionally, SnRK1 plays important roles in plant growth, development and stress adaptations in response to different energy status. Recent studies have demonstrated that SnRK1 is involved in grain development and filling. In rice, the loss‐of‐function *Ossnrk1a* mutant displayed the reduced grain filling rate and low seed setting rate compared to wild‐type plants (Hu *et al*., 2022; Li *et al*., 2022). OsSnRK1a regulates the non‐structural carbohydrates distribution between leaf sheath and panicle to control the grain filling, thereby affecting the grain weight and crop yield (Hu *et al*., 2022).

Some members of the basic helix–loop–helix (bHLH) family have been reported to participate in response to endogenous hormonal and environmental signals and regulation of cell elongation (Jung *et al*., 2016; Oh *et al*., 2014; Pedmale *et al*., 2016). Here, in this study, an atypical bHLH gene, *TabHLH489*, in the 2D chromosome of wheat was identified by genome‐wide association study (GWAS) and map‐based cloning in wheat that controls grain length. The expression of *TabHLH489* in developing grains was found to have a negative correlation with wheat grain length. Knockout of *TabHLH489* and its homologues led to an increased grain length and weight, whereas the overexpression resulted in decreased grain length and weight. Moreover, TaSnRK1α1 interacts with and phosphorylates TabHLH489 to promote its degradation, thus promoting grain development. Additionally, BR decreases the transcript levels of *TabHLH489* through TaBZR1 to promote grain development. Natural variations in the promoter could influence the expression of *TabHLH489* by affecting the binding ability of TaBZR1. These variations may have been selected during the breeding process, implying that natural variations in *TabHLH489* contribute to grain length diversity in wheat and may be a promising candidate for improving wheat yield.

## Results

### Map‐based cloning of *TabHLH489*

**Figure 1:**
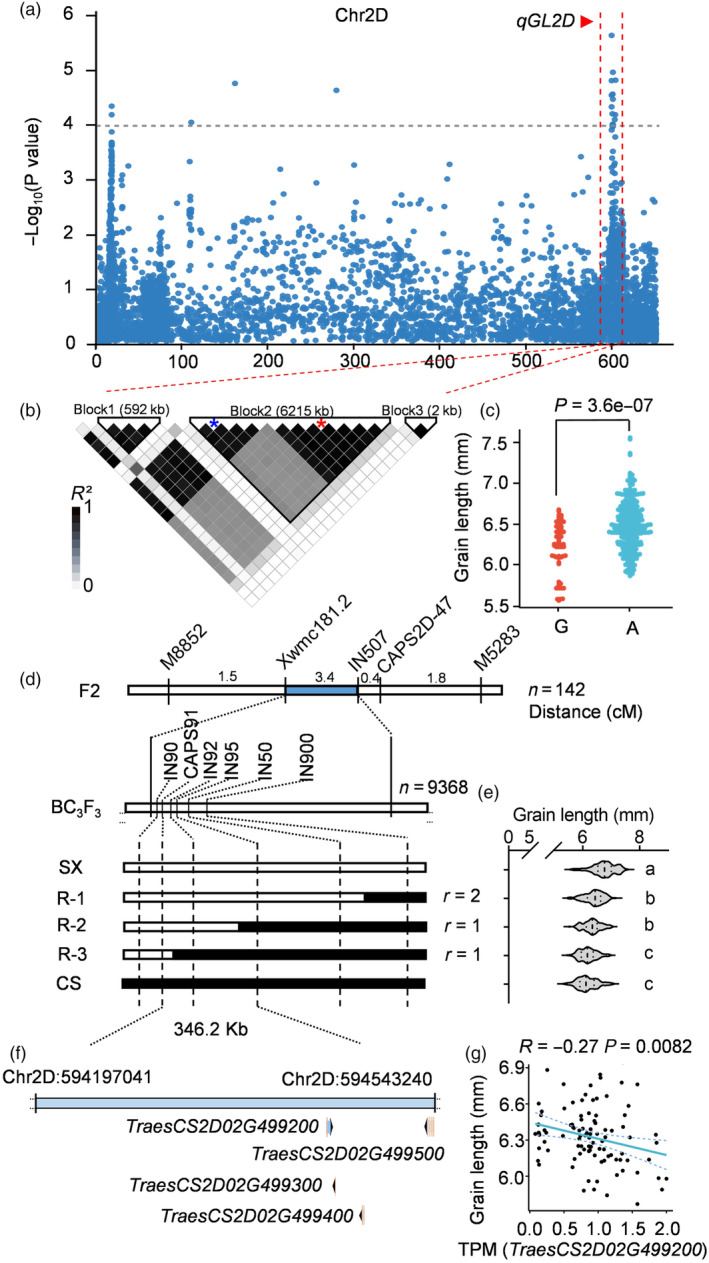
Map‐based cloning of grain length regulating gene *TabHLH489*. (a, b) Manhattan plot (a) and LD heatmap (b) showing the significant peak for wheat grain length on chromosome 2D. The horizontal dashed black line indicates the significance threshold *P* value (*P* = 1.0E−04) for marker‐trait associations. The 10‐Mb region surrounding peak marker of *qGL2D* was indicated by dashed red vertical lines and a LD heatmap for pairwise *R* ^2^ value of markers in this region was shown with white to black represents *R* ^2^ = 0–1. The leading SNP and gene *TabHLH489* were indicated by red and blue asterisks, respectively. (c) The distribution of grain length between haplotypes defined by the leading SNP on chromosome 2D. Significance was determined by Student's *t*‐test (*n* = 364). (d) The candidate gene for grain length was fine‐mapped to a 346.2 Kb region between markers CAPS91 and IN95. Boxs in white and black indicate SX and CS genotype of regions, respectively. ‘*r*’ indicates number of recombinants. Markers' information was listed in Table S2. (e) Progeny testing of homozygous recombinants (BC_3_F_4_) was used to narrow down the candidate region. The grains (*n* > 100) came from six individual plants on average. Different letters above bars indicate statistically significant differences between samples (one‐way ANOVA, *P* < 0.05, *n* ≥ 6). (f) Predicted candidate genes in the narrowed region according to the International Wheat Genome Sequencing Consortium wheat genome (IWGSC, RefSeq V1.1). (g) Association analysis of *TraesCS2D02G499200* expression level and grain length in 10‐DPA seeds of 102 representative wheat varieties. Different lowercase letters indicate statistically significant differences between samples (one‐way ANOVA, *P* < 0.05).

To identify genes associated with grain length, a previously characterized common wheat population composed of 343 cultivars and 21 landraces (Wang *et al*., 2020), capturing the genetic diversity and geographical distribution of the wheat population, were utilized in GWAS with a marker significance threshold *P* value of 1.0E−04 (Figure S1a,b). The locus *qGL2D* on chromosome 2D, evidenced by the leading SNP *AX‐111195248* (2D:599896645, *P* = 2.63E−06), was of particular interest as it accounted for 15.17% of grain length variation in the population (Figure 1a). The associated physical interval of the leading SNP was estimated to be 6.22 Mb (linkage disequilibrium block 2, 594.19 Mb–600.4 Mb) (Figure 1b). The leading SNP was able to differentiate the association panel into two haplotypes and 60 accessions with the favourable A‐type haplotype exhibit longer grain and higher grain weight compared to those with the G‐type haplotype (Figure 1c). To identify the candidate gene in this region, an SSR marker Xwmc181.2 and an InDel marker IN507 were used to construct the BC_3_F_3_ population using two wheat varieties, Chinese spring (CS) of the G‐type and Shixin 828 (SX) of the A‐type, which showed significant differences in grain length and grain weight. Total 9368 individuals of the BC_3_F_3_ population were generated and used to narrow down the locus *qGL2D* associated with grain length. The result of fine mapping analysis indicated that the candidate gene of *qGL2*D is situated within the 346.2 Kb region flanked by markers CAPS91 and IN95, which included a large intergenic region and four genes annotated with high confidence according to IWGSC RefSeq V1.1 (Figure 1d–f). Among these four genes, *TraesCS2D02G499300* and *TraesCS2D02G499400* had notably low expression levels in wheat grains, while *TraesCS2D02G499200* and *TraesCS2D02G499500* displayed high expression levels with significant differences between CS and SX varieties (Figure S1c). Additionally, we compared the expression levels of *TraesCS2D02G499200* and *TraesCS2D02G499500* in 10‐DPA (days post‐anthesis) seeds from 102 wheat varieties, revealing a significant negative correlation (*R* = −0.27, *P* = 0.0082) between the expression of *TraesCS2D02G499200* and grain length (Figure 1g) and a weaker and less significant correlation (*R* = −0.24, *P* = 0.014) between the expression of *TraesCS2D02G499500* and grain length (Figure S1d). It is worth noting that *TraesCS2D02G499500* encodes a homologue of Arabidopsis PELOTA (At4G27650), which is involved in RNA quality control systems during translation (Eberhart and Wasserman, 1995; Szádeczky‐Kardoss *et al*., 2018a,b). In contrast, *TraesCS2D02G499200* encodes an atypical bHLH transcription factor known as TabHLH489 in wheat based on previous research (Wei and Chen, 2018). TabHLH489 is a member of the bHLH subfamily 26, exhibiting high homology with UPB1, AIF1‐AIF4 and IBH1, all of which play roles in inhibiting plant cell elongation (Bai *et al*., 2012; Wei and Chen, 2018; Zhang *et al*., 2009). Therefore, we hypothesized that *TraesCS2D02G499200*, also known as *TabHLH489*, may be the potential candidate gene responsible for controlling grain length.

### TabHLH489 inhibits the grain development

**Figure 2:**
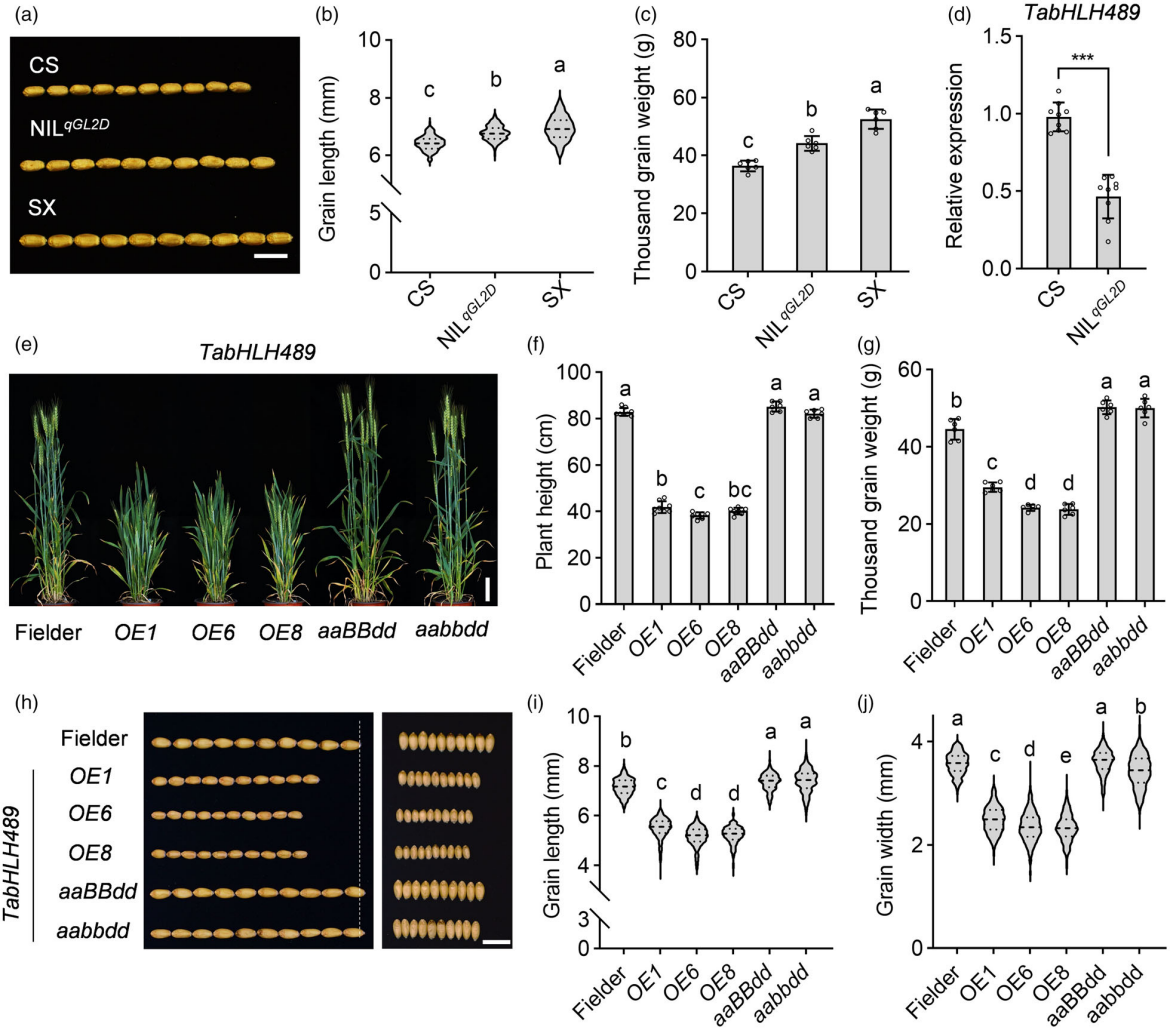
*TabHLH489* negatively regulates wheat grain development. (a–c) Wheat grain morphology of CS, SX and NIL^*qGL2D*^. The grains (*n* > 100) came from six individual plants on average. Scale bar = 1 cm. (d) Quantitative RT‐PCR analysis of the expression level of *TabHLH489* in 5‐DPA seeds of CS and NIL^*qGL2D*^. Error bars indicate ±SD from three biological repeats. *TaADPRF* was used as an internal control. (e, f) Plant architecture of Fielder, *OE‐TabHLH489* and *TabHLH489* knockout plants at the heading stage. Error bars indicate ±SD (*n* ≥ 6). Scale bar = 10 cm. (g–j) Wheat grain morphology of Fielder, *OE‐TabHLH489* and *TabHLH489* knockout plants. The grains (*n* > 300) came from six individual plants on average. Scale bar = 1 cm. Different letters above bars indicate statistically significant differences between samples (one‐way ANOVA, *P* < 0.05). ‘***’ indicates statistically significant differences between samples (Student's *t*‐test, *P* < 0.001).

To validate the role of *qGL2D* in the regulation of grain length, we constructed the near‐isogenic line of *qGL2D* (BC_5_F_2_) which was the substitution line in CS genetic background. Compared to CS with the grain length of 6.43 mm and 1000‐grain weight of 36.42 g, NIL^*qGL2D*^ and SX have large grains with the grain length of 6.78 mm and 6.95 mm and 1000‐grain weight of 44.22 g and 52.54 g, respectively (Figure 2a–c). Quantitative RT‐PCR analysis confirmed that the relative expression level of *TabHLH489* in NIL^*qGL2D*^ was reduced to 46.4% compared to CS in 5‐DPA seeds (Figure 2d). Additionally, *TabHLH489* exhibited varied expression across different stages of wheat development, with the highest expression observed in the third leaf of wheat seedlings (Figure S2a). TabHLH489 was located in the nuclear in tobacco leaves (Figure S2b). To identify whether *TabHLH489* is the key gene of *qGL2D*, which contributes to longer grain length in SX, we generated the knockout mutants of *TabHLH489* gene and its homologous genes on chromosome 2A and/or 2B using CRISPR/Cas9 method in wheat cultivars Fielder background (Figure S3a,b). The knockout mutants showed no difference in plant height compared to Fielder, but had longer and heavier grains than Fielder, suggesting *TabHLH489* plays an important role in wheat grain development (Figures 2e–i, S4a–d). However, the triple mutant *tabhlh489‐aabbdd* showed a slight decrease of grain width compared to Fielder, indicating there may be a negative effect of grain width resulted by knocking out of three homologous *TabHLH489* genes (Figure 2h,j). Although the expression level of *TabHLH489* was negatively correlated with grain length in 102 wheat varieties, the protein sequence alignment of TabHLH489 (from CS) and TabHLH489‐S (from SX) revealed 8 amino acid substitutions and 3 amino acid insertions in TabHLH489‐S compared to TabHLH489 (Figure S5). To examine whether these CDS variations are involved in the grain length regulation in the *qGL2D*, the transgenic plants performing similar expression levels of *TabHLH489* or *TabHLH489‐S* were selected for further phenotypic analysis (Figure S6a). The *pUbi:TabHLH489‐YFP* (*OE‐TabHLH489*) and *pUbi:TabHLH489‐S‐YFP* (*OE‐TabHLH489‐S*) transgenic plants both displayed significantly decreased plant height, grain length and grain weight compared to Fielder (Figures 2e–i, S6b–f). These results suggested that the difference in grain length between CS and SX was probably not due to the coding sequence differences between *TabHLH489* and *TabHLH489‐S* (Figure S5), but rather may be attributed to the different promoter sequences of *TabHLH489* in CS and SX, which results in varying expression levels of *TabHLH489* in these two varieties (Figures S1c, S7 and S8). Together, these results indicated that TabHLH489 is a negative regulator of wheat grain development.

### TabHLH489 reduces the grain length by inhibiting cell elongation

**Figure 3:**
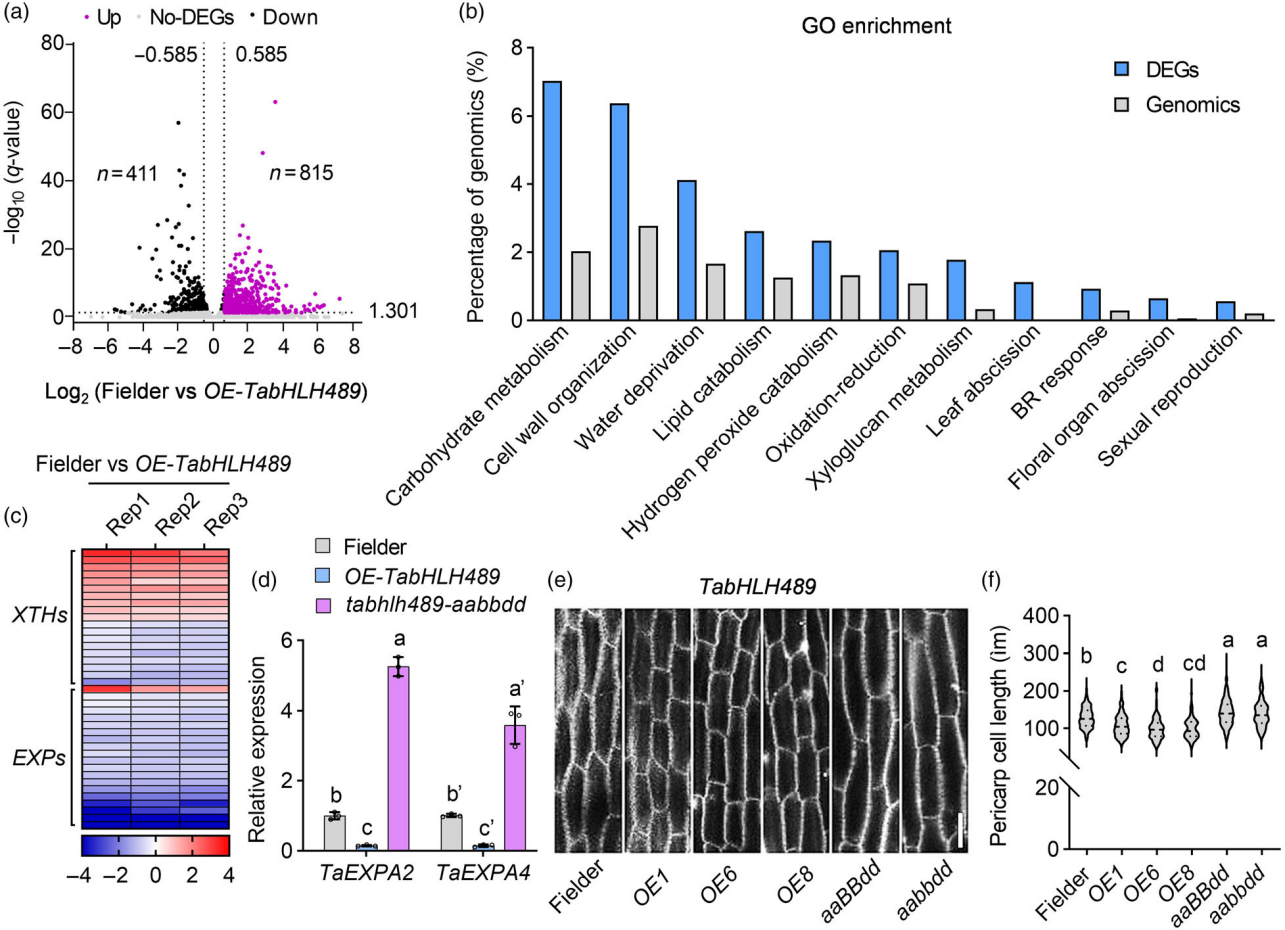
*TabHLH489* inhibits wheat pericarp cell elongation. (a) Differentially expressed genes (DEGs) in seeds of Fielder and *OE‐TabHLH489* at 5‐DPA stage. (b) GO analysis of DEGs regulated by *TabHLH489*. (c) Heatmap analysis of cell wall development related genes *Xyloglucan endotransglucosylase/hydrolases* (*XTHs*) and *Expansins* (*EXPs*) regulated by *TabHLH489* in 5‐DPA seeds. (d) Quantitative RT‐PCR analysis the expression of *TaEXPA2* and *TaEXPA4* in 5‐DPA seeds of Fielder, *OE‐TabHLH489* and *tabhlh489‐aabbdd* mutant. Error bars indicate ±SD (*n* = 3). *TaADPRF* was used as an internal control. (e, f) The grain pericarp cell length of 15‐DPA seeds from Fielder, *OE‐TabHLH489* and *TabHLH489* knockout plants. The cells (*n* > 100) came from six individual plants on average. Scale bar = 50 μm. Different letters above bars indicates statistically significant differences between samples (two‐way ANOVA [d], one‐way ANOVA [f], *P* < 0.05).

To further investigate the functional mechanism of TabHLH489 in wheat grain development, we performed the transcriptomic analysis using 5‐DPA seeds of Fielder and *OE‐TabHLH489* plants. Overall, we identified 1226 differentially expressed genes, with 815 upregulated and 411 downregulated by more than 1.5‐fold in *OE‐TabHLH489* compared to Fielder plants (Figure 3a, Table S1). Gene ontology (GO) analysis showed that genes involved in carbohydrate metabolism, cell wall organization, water deprivation, lipid catabolism, oxidation–reduction process, xyloglucan catabolism and BR response were highly enriched in TabHLH489‐regulated genes (Figure 3b). Cell wall organization is critical for the cell elongation, suggesting that *TabHLH489* may regulate wheat grain development by modulating cell elongation. Remarkably, two family genes, *Xyloglucan endotransglucosylase/hydrolases* (*XTHs*) and *Expansins* (*EXPs*), which were related to cell elongation, performed significantly different expression profiles in *OE‐TabHLH489* and Fielder (Figure 3c). Quantitative RT‐PCR analysis further confirmed that the expression levels of cell elongation‐related genes *TaEXPA2* (*TraesCS3A02G344800*) and *TaEXPA4* (*TraesCS1D02G299700*) in 5‐DPA seeds were significantly decreased in *OE‐TabHLH489* plants, while increased in *tabhlh489‐aabbdd* triple mutant (Figures 3d, S6g). Consistent with this, histological sectioning analysis showed that the *tabhlh489‐aaBBdd* double mutant and *tabhlh489‐aabbdd* mutant had significantly enlarged cell length of grain pericarp, while the overexpression of *TabHLH489* or *TabHLH489‐S* both displayed the reduced cell length (Figures 3e,f, S6h,i). These results indicated that TabHLH489 regulates the wheat grain length by repressing the expression of cell elongation‐related genes in early grain developing stage.

### TabHLH489 interacts with TaSnRK1α1 to regulate grain development

**Figure 4:**
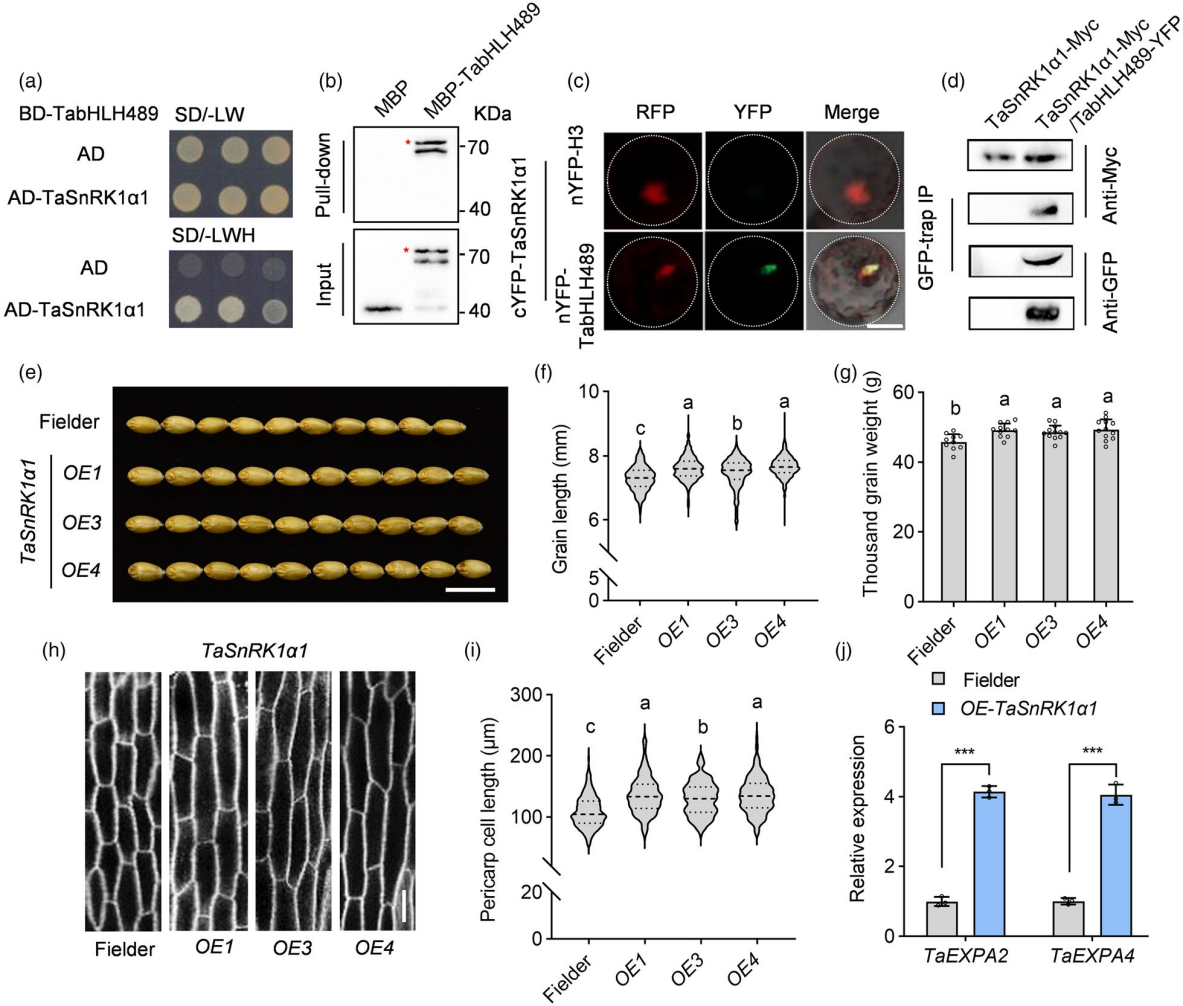
TaSnRK1α1 interacts with TabHLH489 and promotes wheat grain development. (a) TaSnRK1α1 interacts with TabHLH489 in yeast. (b) TaSnRK1α1 directly interacts with TabHLH489 *in vitro*. Glutathione agarose beads loaded with GST‐TaSnRK1α1 were incubated with equal amounts of MBP or MBP‐TabHLH489. Proteins bound to GST‐TaSnRK1α1 were detected by immunoblot analysis with an anti‐MBP antibody. The red asterisk indicates the MBP‐TabHLH489 protein band. (c) Confocal images of ratiometric bimolecular fluorescence complementation assays showed that TaSnRK1α1 interacts with TabHLH489 in protoplast. nYFP‐H3 indicates nYFP‐Histone3. Scale bar = 20 μm. (d) TaSnRK1α1 interacts with TabHLH489 in plants. Immunoprecipitation was performed using the tobacco leaves transient expressing *p35S:TaSnRK1α1‐Myc* only or co‐expressing *p35S:TaSnRK1α1‐Myc* and *p35S*:TabHLH489*‐YFP*. The co‐immunoprecipitation experiments were performed using GFP‐Trap agarose beads and the immunoblots were probed with anti‐Myc or anti‐GFP antibodies. (e–g) Wheat grain morphology of Fielder and *TaSnRK1α1* overexpression lines. The grains (*n* > 300) came from six individual plants on average. Scale bar = 1 cm. (h, i) The grain pericarp cell length of Fielder and *OE‐TaSnRK1α1* plants. The cells (*n* > 100) came from six individual plants on average. Scale bar = 50 μm. (j) Quantitative RT‐PCR analysis of the expression of *TaEXPA2* and *TaEXPA4* in 5‐DPA seeds of Fielder and *OE‐TaSnRK1α1* plants. Error bars indicate ±SD (*n* = 3). *TaADPRF* was used as an internal control. Different letters above bars indicate statistically significant differences between samples (one‐way ANOVA, *P* < 0.05). ‘***’ indicates statistically significant differences between samples (Student's *t*‐test, *P* < 0.001).

To investigate the functional mechanism of TabHLH489 in wheat grain development, we performed a yeast two‐hybrid (Y2H) screen to identify interaction proteins using the full length TabHLH489 protein as bait and the Y2H complementary DNA library that was constructed with different organs of wheat, including root, leaf, young spike and seeds of different days post anthesis. Among the putative TabHLH489‐interacting proteins, TraesCS1D02G353300 exhibited the strong interaction with TabHLH489. *TraesCS1D02G353300* is an orthologous gene of the catalytic α‐subunit of SnRK1 in wheat, thus was named as *TaSnRK1α1*. SnRK1 plays critical roles in gain filling and development in rice (Hu *et al*., 2022; Li *et al*., 2022). We selected TaSnRK1α1 for further research to reveal the mechanism by which the TaSnRK1α1‐TabHLH489 module regulates grain development. Additional Y2H assays confirmed that TabHLH489 interacted with TaSnRK1α1 in yeast (Figure 4a). The *in vitro* protein pull‐down assays revealed that glutathione S‐transferase (GST) fusion protein GST‐TaSnRK1α1 interacted with maltose binding protein (MBP) fusion protein MBP‐TabHLH489, but not with MBP alone (Figure 4b). To determine whether TaSnRK1α1 interacts with TabHLH489 in planta, we performed transient ratiometric bimolecular fluorescence complementation (rBiFC) assays in the mesophyll protoplast of leaves. The strong yellow fluorescent signals were observed in the protoplast co‐transformed with TaSnRK1α1‐cYFP and TabHLH489‐nYFP, but not in those co‐transformed with TaSnRK1α1‐cYFP and Histone3‐nYFP (Figure 4c). Co‐immunoprecipitation (Co‐IP) assays using *A. tumefaciens*‐mediated transient infiltration in *N. benthamiana* expressing *p35S:TanRK1α1‐Myc* only or co‐expressing *p35S:TaSnRK1α1‐Myc* and *p35S:TabHLH489‐YFP* further confirmed the interaction between TaSnRK1α1 and TabHLH489 in planta (Figure 4d). Together, these results indicated that TaSnRK1α1 interacts with TabHLH489 *in vitro* and *in vivo*.

To investigate the potential role of TaSnRK1α1 in wheat grain development, we generated the *TaSnRK1α1* overexpression plants (*pUbi:TaSnRK1α1‐YFP, OE‐TaSnRK1α1*) and the triple knockout mutants of *TaSnRK1α1* (situated on chromosome 1D) and its two homologues *TaSnRK1α1‐1A*, *TaSnRK1α1‐1B* (*tasnrk1α1‐ko*) in Fielder background (Figure S9a–f). The overexpression of *TaSnRK1α1* led to increased grain length and grain weight (Figures 4e–g, S9b,c), as well as longer cell length in grain pericarps compared to Fielder plants (Figure 4h,i). The *tasnrk1α1‐ko* mutants displayed no significant difference in grain length and grain pericarp cell length, likely due to the redundancy of 15 *TaSnRK1* homologue genes in wheat genome (Figure S9g–j). Furthermore, quantitative RT‐PCR analysis showed that the overexpression of *TaSnRK1α1* strikingly increased the transcript levels of *TaEXPA2* and *TaEXPA4* in 5‐DPA seeds (Figure 4j). These results suggested that TaSnRK1α1 acts as a positive regulator of wheat grain development.

### TaSnRK1α1 phosphorylates TabHLH489 to promote its degradation

**Figure 5:**
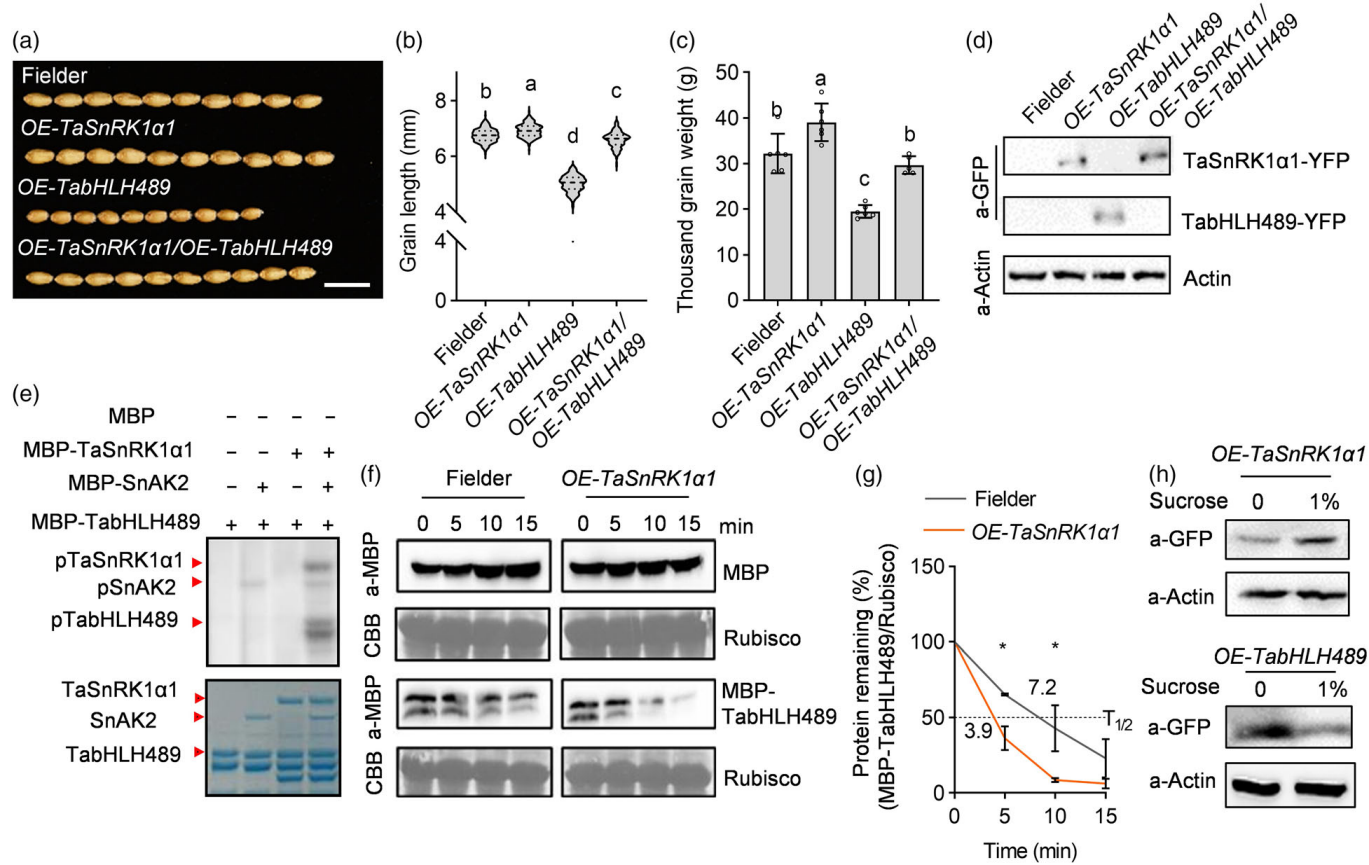
TaSnRK1α1 phosphorylates and destabilizes TabHLH489. (a–c) Wheat grain morphology of Fielder, *OE‐TaSnRK1α1, OE‐TabHLH489* and the progenies derived from a cross between *OE‐TaSnRK1α1* and *OE‐TabHLH489* plants. The grains (*n* > 100) came from more than five individual plants on average. Scale bar = 1 cm. (d) Immunoblot assay of the protein levels of TabHLH489 and TaSnRK1α1 in Fielder, *OE‐TabHLH489*, *OE‐TaSnRK1α1* and *OE‐TaSnRK1α1/OE‐TabHLH489* plants. TaActin was used as the loading control. (e) TaSnRK1α1 phosphorylates TabHLH489 *in vitro*. Upper image was the gel containing proteins labelled with ATP‐γ‐^32^p, while the following image was the gel staining with coomassie brilliant blue. (f, g) Degradation of MBP and MBP‐TabHLH489 in cell‐free system from Fielder and *OE‐TaSnRK1α1* extracts. Recombinant purified MBP and MBP‐TabHLH489 were added to the protein extracts and incubated at 22 °C for different times. Protein abundance was evaluated using an anti‐MBP antibody. The band intensity was quantified by ImageJ. (h) Sucrose promoted TaSnRK1α1 accumulation but induced TabHLH489 degradation. Total proteins were extracted from 7‐day‐old seedlings of *OE‐TaSnRK1α1* and *OE‐TabHLH489* plants after a 6‐h treatment with 1% sucrose under light conditions. Different letters above bars indicate statistically significant differences between samples (one‐way ANOVA, *P* < 0.05). ‘*’ indicate statistically significant differences between samples (Student's *t*‐test, *P* < 0.05).

To examine the impact of the interaction between TaSnRK1α1 and TabHLH489, we generated the progeny from a cross between *OE‐TaSnRK1α1* and *OE‐TabHLH489* plants. Phenotypic analysis revealed that grains from the crossed progeny, as well as pericarp cell length, were longer than those from *OE‐TabHLH489* plants. Additionally, grain weight in the crossed progeny was recovered compared to Fielder, suggesting that TabHLH489 activity was partially inhibited by TaSnRK1α1 (Figures 5a–c, S10a–c). Quantitative RT‐PCR analysis showed that the expression levels of *TabHLH489* in 5‐DPA seeds of the *OE‐TaSnRK1α1/OE‐TabHLH489* plants were similar to those of *OE‐TabHLH489* plants (Figure S10a). Nevertheless, the results of the western blot analysis revealed that the protein levels of TabHLH489 were significantly reduced in the *OE‐TaSnRK1α1/OE‐TabHLH489* plants compared to *OE‐TabHLH489* plants (Figure 5d), suggesting that the overexpression of TaSnRK1α1 results in the decreased protein stability of TabHLH489.

Considering that TaSnRK1α1 is an evolutionarily conserved energy sensor kinase and TabHLH489 interacts with TaSnRK1α1, the kinase assays were performed to determine whether TabHLH489 is the substrate of TaSnRK1α1. The results showed that MBP‐TaSnRK1α1 alone failed to phosphorylate MBP‐TabHLH489. However, in the presence of the upstream kinase MBP‐SnAK2, MBP‐TaSnRK1α1 is activated by SnAK2‐mediated phosphorylation. Activated MBP‐TaSnRK1α1 phosphorylated MBP‐TabHLH489, but not MBP only (Figure 5e). To analyse the effects of TaSnRK1α1‐mediated phosphorylation on the function of TabHLH489, we investigated the degradation rate of MBP‐TabHLH489 in cell‐free extracts of *OE‐TaSnRK1α1* and wild type plants. The result showed that the degradation rate of MBP‐TabHLH489 was 1.85 times faster in the extracts derived from *OE‐TaSnRK1α1* compared to those obtained from Fielder, as determined by the half‐life time (*T*_1/2_) of MBP‐TabHLH489, suggesting that TaSnRK1α1‐mediated phosphorylation reduces the protein stability of TabHLH489 (Figure 5f,g). Moreover, the sucrose treatment promoted the accumulation of TaSnRK1α1, but decreased the protein stability of TabHLH489 (Figure 5h). Taken together, TaSnRK1α1 promotes wheat grain development through decreasing TabHLH489 protein stability by phosphorylation.

### TabHLH489 reduces the BR sensitivity of wheat

**Figure 6:**
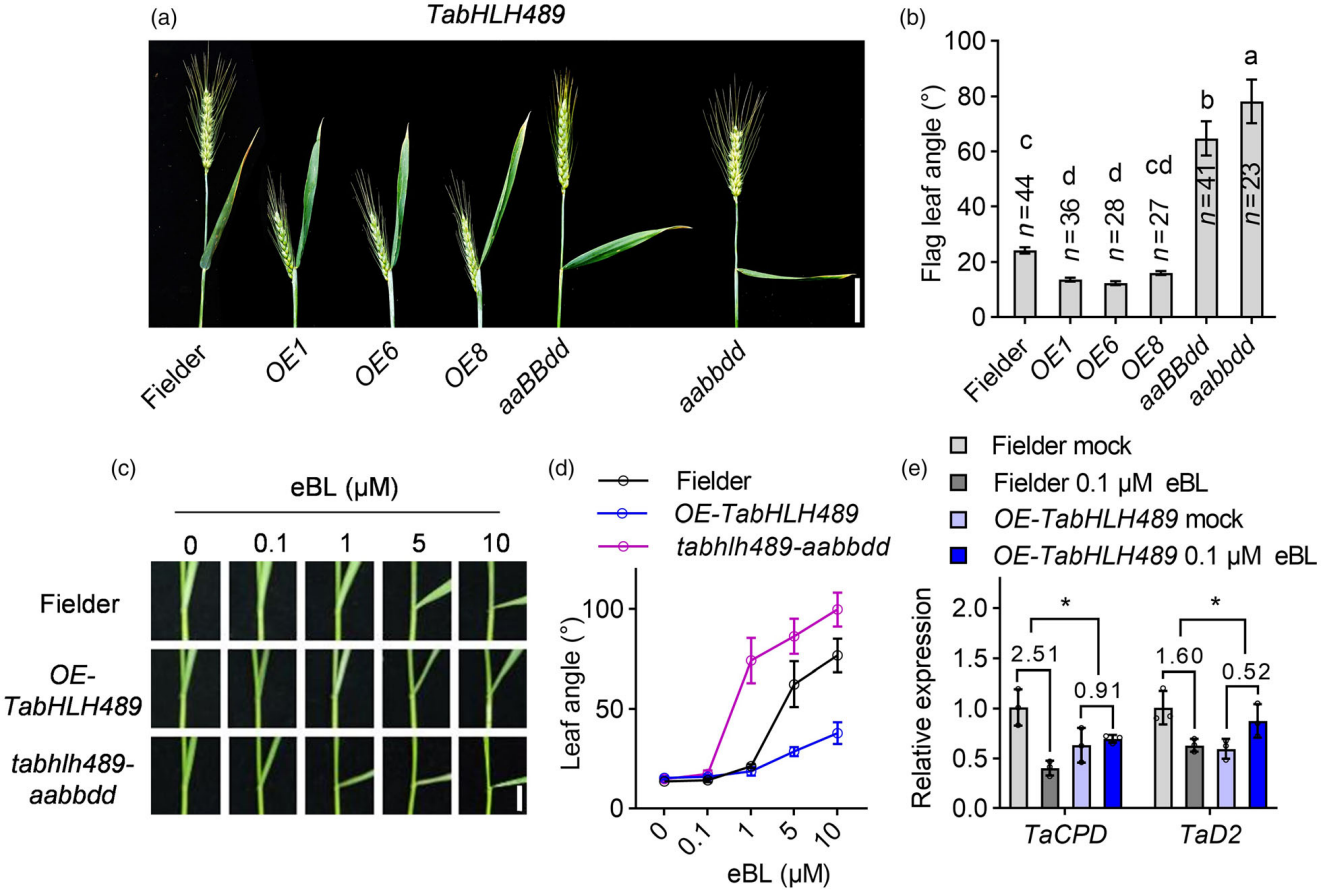
TabHLH489 reduces the BR sensitivity of wheat. (a, b) The flag leaf angles of Fielder, *OE‐TabHLH489* and *TabHLH489* knockout mutants. Error bars indicate ±SE, scale bar = 5 cm. (c, d) The leaf angles of Fielder, *OE‐TabHLH489 and tabhlh489‐aabbdd* mutant in the presence of different concentrations of eBL. Error bars indicate ±SE, scale bar = 5 cm. (e) Quantitative RT‐PCR analysis of the expression level of *TaCPD and TaD2* in 7‐day‐old Fielder, *OE‐TabHLH489* plants with or without 100 nM eBL foliar‐sprayed treatment for 3 h. Error bars indicate ±SD from three biological repeats. *TaADPRF* was used as an internal control. Different lowercase letters indicate statistically significant differences between samples (one‐way ANOVA, *P* < 0.05). ‘*’ indicate statistically significant differences between samples (Student's *t*‐test, *P* < 0.05).

The angle of wheat leaf inclination between leaf blade and the culm is an important agronomic trait and contributes to the wheat architecture and grain yields. BR plays a unique and crucial role in determine leaf inclination (Gao *et al*., 2019; Min *et al*., 2019; Wu *et al*., 2016). The overexpression of *TabHLH489* or *TabHLH489‐S* both resulted in the decreased flag leaf inclination, whereas *tabhlh489‐aaBBdd* and *tabhlh489‐aabbdd* mutants both showed the significant increased flag leaf inclination (Figures 6a,b, S6j,k). The dwarfism phenotype and erect leaves of *TabHLH489* and *TabHLH489‐S* overexpression plants led us to speculate that TabHLH489 may be a negative regulator of BR signalling pathway. To test this hypothesis, we analysed the BR sensitivity of *tabhlh489‐aabbdd* mutant and *TabHLH489* overexpression lines. BR treatment increased the leaf inclination of Fielder plants in a dose‐dependent manner, while such promoting effects were enhanced in *tabhlh489‐aabbdd* mutant, but reduced in *OE‐TabHLH489* plants (Figure 6c,d). Quantitative RT‐PCR analysis showed that BR treatment significantly reduced the expression of *TaCPD* and *TaD2* in Fielder plants, but had weak effects in *OE‐TabHLH489* plants (Figure 6e). These results indicated that TabHLH489 functions as a negative regulator of BR signalling in wheat.

### BR induces the wheat grain length by repressing *TabHLH489* expression

**Figure 7:**
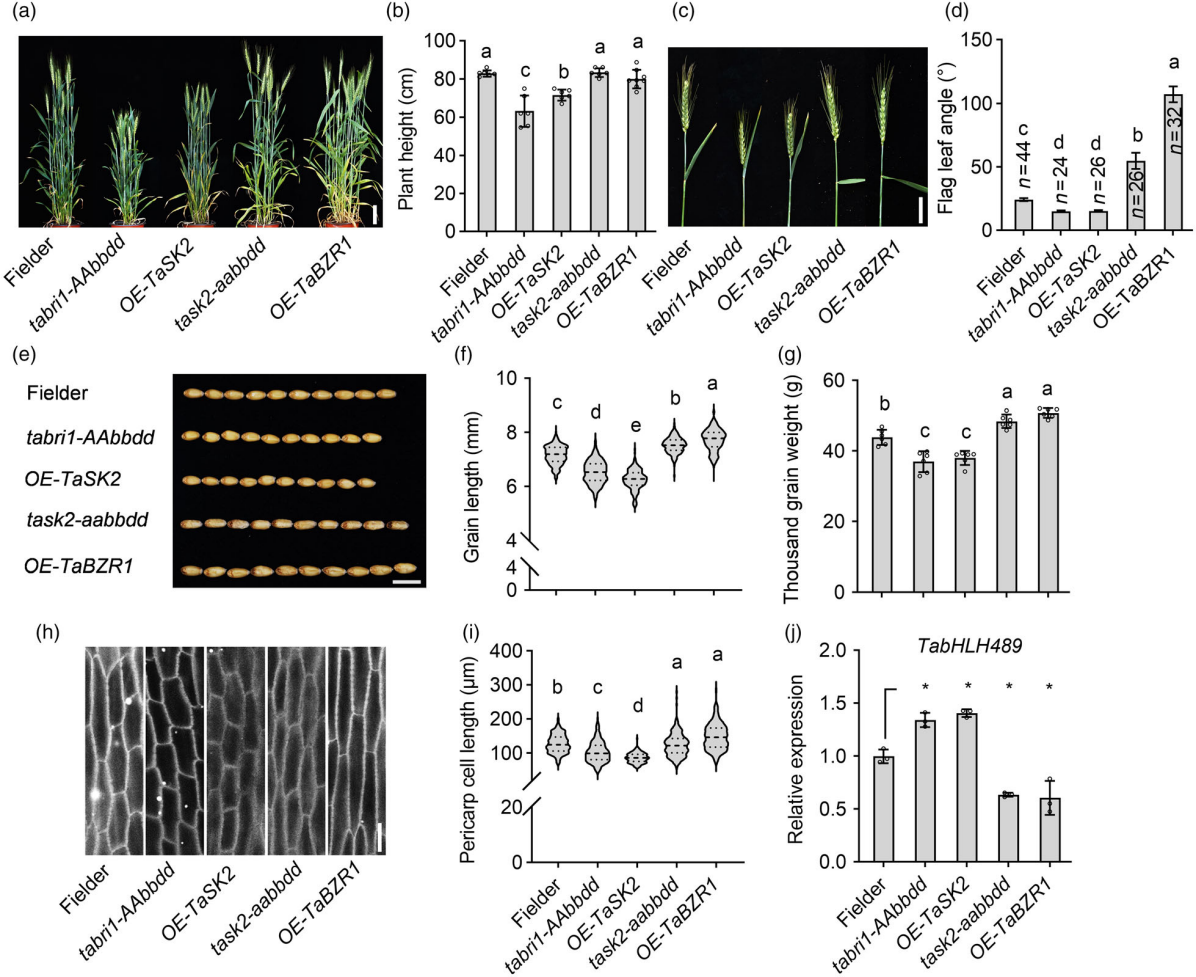
BR induces wheat grain length by repressing *TabHLH489* expression. (a, b) Plant architecture of Fielder, *tabri1‐AAbbdd*, *OE‐TaSK2*, *task2‐aabbdd* and *OE‐TaBZR1* at the heading stage. Error bars indicate ±SD (*n* ≥ 6). Scale bar = 10 cm. (c, d) The flag leaf angles of Fielder, *tabri1‐AAbbdd*, *OE‐TaSK2*, *task2‐aabbdd* and *OE‐TaBZR1*. Error bars indicate ±SE, Scale bar = 5 cm. (e–g) Wheat grain morphology of Fielder, *tabri1‐AAbbdd*, *OE‐TaSK2*, *task2‐aabbdd* and *OE‐TaBZR1*. The grains (*n* > 100) came from six individual plants on average. Scale bar = 1 cm. (h, i) The grain pericarp cell length of 15‐DPA seeds from Fielder, *tabri1‐AAbbdd*, *OE‐TaSK2*, *task2‐aabbdd* and *OE‐TaBZR1*. The cells (*n* > 100) came from six individual plants on average. Scale bar = 50 μm. (j) Quantitative RT‐PCR analysis of *TabHLH489* expression in 5‐DPA seeds of Fielder, *tabri1‐AAbbdd*, OE‐*TaSK2*, *task2‐aabbdd* and *OE‐TaBZR1* plants. Error bars indicate ±SD (*n* = 3). *TaADPRF* was used as an internal control. Different lowercase letters indicate statistically significant differences between samples (one‐way ANOVA, *P* < 0.05). ‘*’ indicate statistically significant differences between samples (Student's *t*‐test, *P* < 0.05).

Considering the critical roles of BR in grain development and lamina joint morphogenesis and the negative regulation of wheat grain size and leaf angle by TabHLH489, we speculated that BR may regulate wheat grain development through modulating *TabHLH489* expression levels. Exogenous BR treatment significantly reduced *TabHLH489* expression and this inhibitory effect became more pronounced with the increase of treatment time (Figure S11). To further test this hypothesis, we generated a series of BR‐related wheat materials. Based on the sequence similarity analysis, the homologous genes of *BRI1, BIN2* and *BZR1* in wheat were identified, including *TaBRI1, TaSK2* and *TaBZR1*. We generated the knockout mutants of *tabri1‐aaBBdd, tabri1‐AAbbdd, tabri1‐aabbDD* (the triple mutant of *tabri1‐aabbdd* is unable to survive) (Figure S12a) and *task2‐aabbdd* (the triple mutant of *TaSK2* [from chromosome 1B] and its two homologues *TaSK2‐1A* and *TaSK2‐1D*) (Figure S13a) using the CRISPR/Cas9 method and the overexpression of *TaSK2* (*OE‐TaSK2*) (Figure S13b) and *TaBZR1* (*OE‐TaBZR1*) (Figure S14a) by expressing the *pUbi:TaSK2‐YFP* and *pUbi:TaBZR1‐YFP* in Fielder background. Phenotypic analysis showed that loss‐of‐function mutants *tabri1‐aaBBdd, tabri1‐AAbbdd, tabri1‐aabbDD* and the overexpression of *TaSK2* caused the dwarf wheat, decreased flag leaf angle, short grains and decreased grain weight compared to Fielder (Figures 7a–g, S12b–h and S13c–i). In contrast, the *task2‐aabbdd* mutant and *TaBZR1* overexpression lines displayed the similar plant height, increased flag leaf angle, longer grains and increased grain weight compared to Fielder (Figures 7a–g, S13c–i and S14b–h). Histologic sectioning analysis showed that *tabri1‐aaBBdd, tabri1‐AAbbdd, tabri1‐aabbDD* and *OE‐TaSK2* plants displayed the reduced cell length of grain pericarps, but *task2‐aabbdd* and *OE‐TaBZR1* plants showed that increased cell length of grain pericarps (Figures 7h,i, S12i,j, S13j,k and S14i,j). These results indicated that BR promotes grain development in wheat. Next, we analysed the expression levels of *TabHLH489* expression levels in 5‐DPA seeds of different BR‐related materials. Quantitative RT‐PCR analysis showed that the transcript levels of *TabHLH489* is significantly increased in 5‐DPA seeds of *tabri1‐AAbbdd* mutant and *OE‐TaSK2* lines, but decreased in 5‐DPA seeds of *task2‐aabbdd* mutant and *TaBZR1* overexpression transgenic plants (Figure 7j). Furthermore, the transcript levels of *TaEXPs* are significantly increased in 5‐DPA seeds of *OE‐TaBZR1* plants (Figure S14k). Furthermore, we examined the expression levels of *TaBRI1*, *TaSK2*, *TaEXPA2* and *TaEXPA4* in 7‐day‐old seedlings of Fielder and *TabHLH489* single, double and triple knockout mutants. The findings revealed that the knockout of *TabHLH489* led to the downregulation of *TaBRI1* and *TaSK2*, as well as the upregulation of *TaEXPA2* and *TaEXPA4*, (Figure S15a,b), indicating that TabHLH489 may regulate wheat grain development in a dose‐dependent manner. These results suggested that BR might promote wheat grain length by inhibiting *TabHLH489* expression.

### DNA variations in the *TabHLH489* promoter confer grain length variations

**Figure 8:**
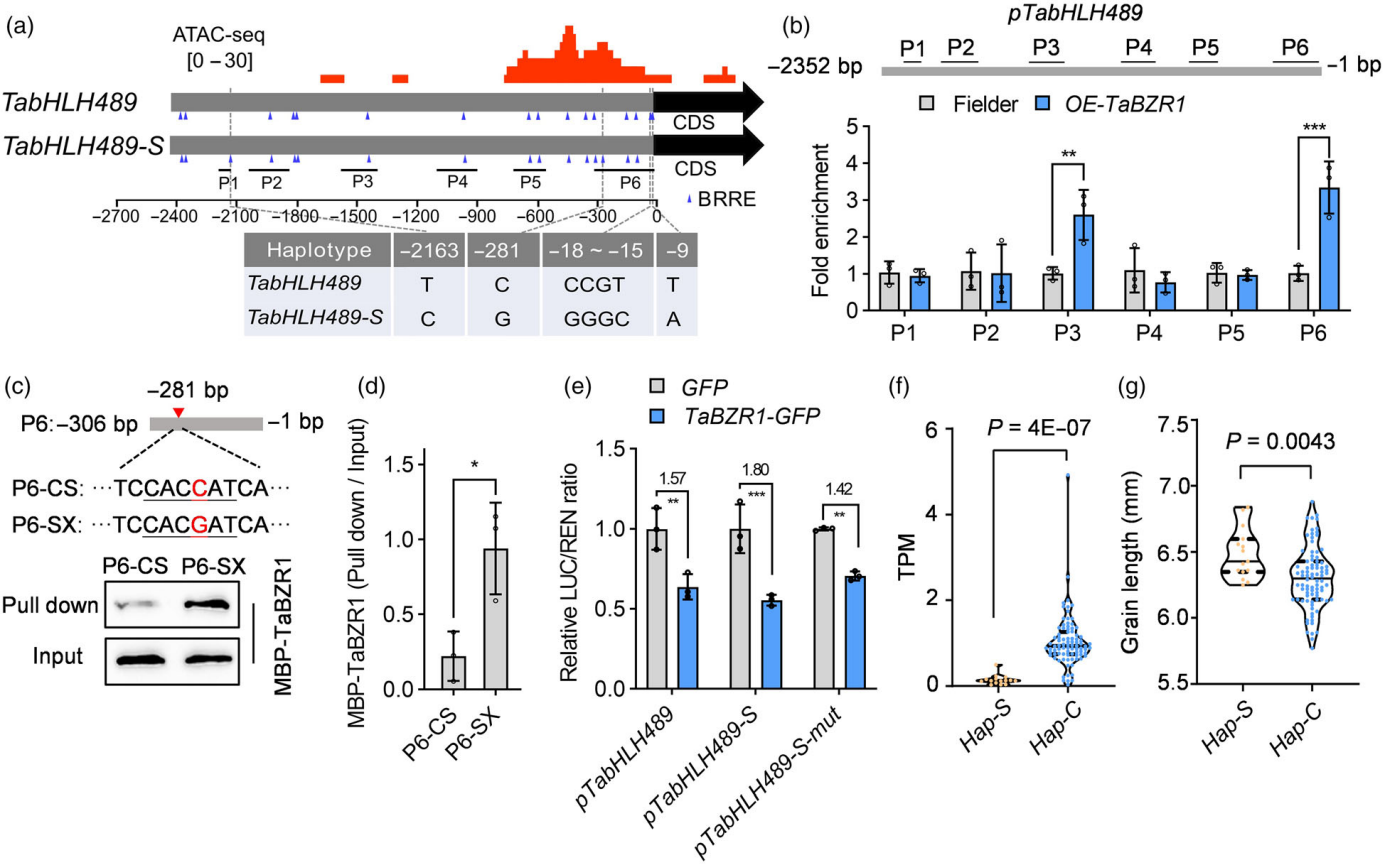
Natural variation in the promoter of *TabHLH489* confer grain length variation. (a) A schematic diagram showing the natural variation, putative BRRE motifs (Blue triangles) and open chromatin regions (Red peaks) in the promoter of *TabHLH489*. *TabHLH489* and *TabHLH489‐S* indicate genes from wheat CS and SX, respectively. ‘[0–30]’ indicates the range of ATAC peaks (Reads Per Kilobase per Million mapped reads, RPKM). Grey boxes indicate the promoter region. Black boxes with arrows indicate the coding sequence. (b) TaBZR1 directly binds to the promoter of *TabHLH489*. The enrichment value was calculated as the ratio between Fielder and *OE‐TaBZR1*. Promoter of *TaADPRF* was used as reference control. Error bars indicate ±SD (*n* = 3). (c, d) DNA‐protein pull down assay showed the different binding ability of TaBZR1 to P6 fragment from CS and SX. Error bars indicate ±SD (*n* = 3). (e) The mutation in P6‐SX (*pTabHLH489‐S‐mut*, containing TC*CAaaAT*CA) declines the TaBZR1's inhibition of *pTabHLH489‐S*. Wheat protoplasts were transformed with dual luciferase reporter constructs containing *pTabHLH489:LUC*, *pTabHLH489‐S:LUC*, *pTabHLH489‐S‐mut:LUC* and/or constructs overexpressing the effector *TaBZR1‐GFP*. LUC (Firefly luciferase) activity was normalized to REN (Renilla luciferase) and the LUC/REN ratios of control samples were normalized to one. Error bars indicate ±SD (*n* = 3). (f) Expression difference of the haplotype *TabHLH489‐S* (*Hap‐S*) or *TabHLH489* (*Hap‐C*) in total 102 representative varieties. (g) Grain length variance of 102 representative varieties containing *Hap‐S* or *Hap‐C*. ‘*’, ‘**’ and ‘***’ indicate statistically significant differences between samples (Student's *t*‐test, *P* < 0.05, *P* < 0.01 and *P* < 0.001, respectively).

BZRs are the key transcription factors in BR signalling and BRRE motif (CGTGT/CG) is responsible for the BZR1 binding. Genomic analysis revealed that *TabHLH489* has an identical genetic sequence in two wheat varieties, CS and Fielder, but has different genetic sequence in SX variety. Motif searching revealed the promoter region of *TabHLH489* underwent DNA variation, causing four putative BRRE motifs to change in both CS and SX (Figures 8a, S16). Among these, SNP‐281 caused a BRRE motif change in the promoter of *TabHLH489* in CS, located in a chromatin‐accessible peak at −500 ~ −272 bp during 6‐DPA of CS endosperm (Figure 8a). This suggests that TaBZR1 may directly regulate *TabHLH489* expression by binding to the *TabHLH489* promoter, resulting in differential expression of *TabHLH489* in CS and SX. The chromatin immunoprecipitation (ChIP)‐qPCR assay with *OE‐TaBZR1* transgenic wheat further showed that TaBZR1 directly bound to *TabHLH489* promoter fragments P3 and P6 (Figure 8b). Analysis of the P6 region in CS and SX revealed a typical BZR1‐binding motif in SX and an atypical BZR1‐binding motif in CS due to one SNP. TaBZR1 exhibited a higher binding ability to the P6 region of SX than CS in DNA protein pull‐down assays (Figure 8c,d). The transient expression assays showed a significant decrease in TaBZR1 transfected luciferase activity derived from *pTabHLH489:LUC* and *pTabHLH489‐S:LUC*, which was significantly reduced by point mutation of TaBZR1‐binding motif in the P6 region of *TabHLH489‐S* promoter (Figure 8e). In summary, TaBZR1 contributes to the differential expression patterns of *TabHLH489* in CS and SX.

Next, we wonder how the different haplotypes of *TabHLH489* could impact its function and regulation of grain length. To investigate the correlation between the SNP at the TaBZR1 binding site with *TabHLH489* expression level in 10‐DPA grains at the population level, we developed a PCR marker targeting the P6 region and genotyped a core collection consisting of 102 representative varieties from the GWAS panel. Compared to the *TabHLH489* haplotype (*Hap‐C*), 15 accessions of *TabHLH489‐S* haplotype (*Hap‐S*) had significantly longer grain and remarkably lower expression level (Figure 8f,g). These findings indicate that natural variations in the proximal (P6) region may contribute to the *TabHLH489* expression differences and grain length variations within wheat population, potentially by affecting binding of TaBZR1.

### Breeding selection of TabHLH489

**Figure 9:**
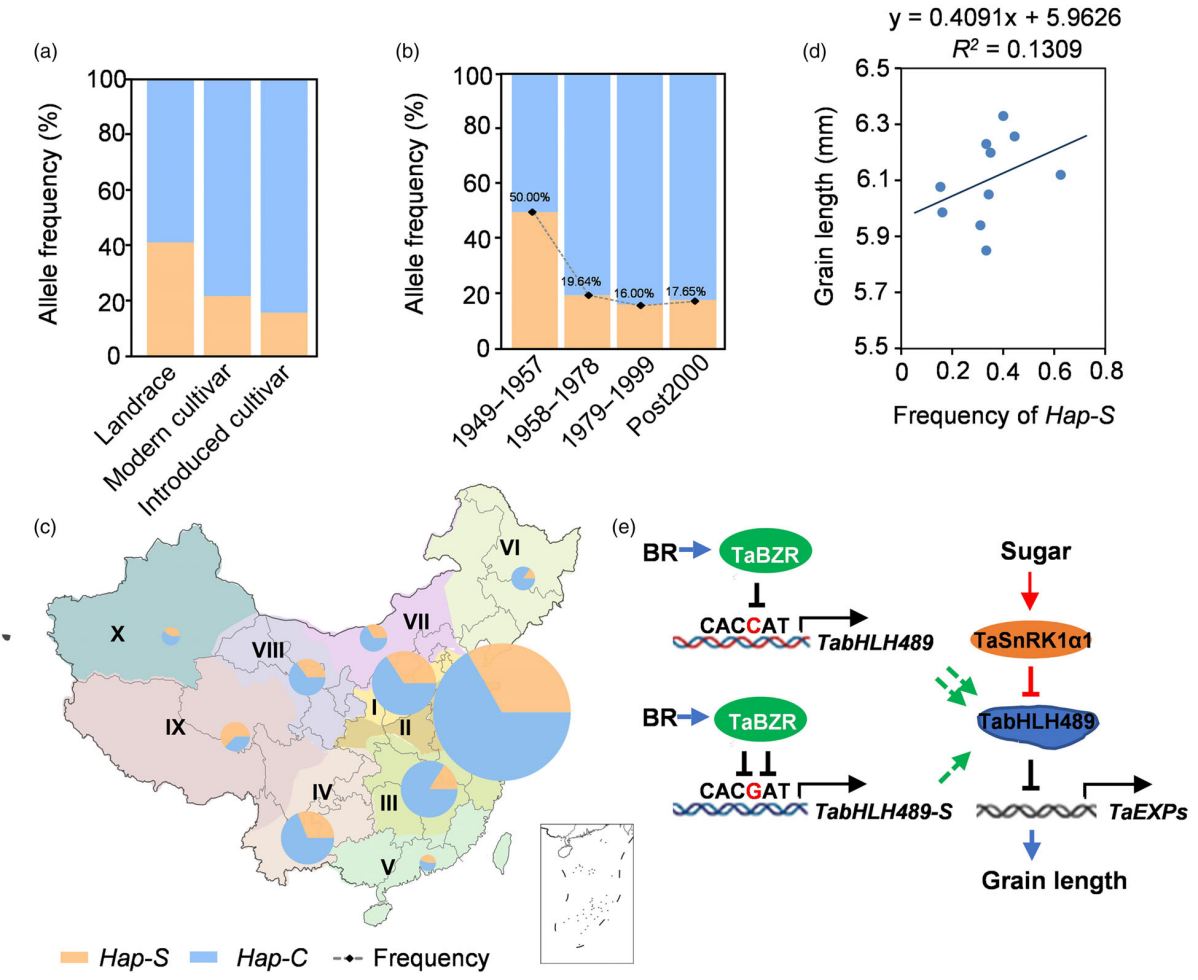
Breeding selection of *TabHLH489* and a working model of BR‐TabHLH489 regulating grain length. (a, b) The frequency of two haplotypes in different category (a) and breeding periods (b) in wheat mini core collections (*n* = 287). (c) Distribution of *Hap‐S* and *Hap‐C* in the major Chinese agro‐ecological zones. The size of pie charts in the geographical map showing the number of cultivars, with percentage of two haplotypes in different colours. (d) Correlation of grain length with the frequency of *Hap‐S* in 10 ecological zones. (e) A working model of TabHLH489 and TaSnRK1α1 integrating BR and sugar signals to regulate wheat grain length. TabHLH489 acts as a negative regulator of wheat grain length by suppressing the expression of cell‐elongation genes *TaEXP2* and *TaEXP4*. TaSnRK1α1 interacts with and phosphorylates TabHLH489 to promote its degradation, thereby facilitating grain development. Sugar induces the accumulation of TaSnRK1α1 but promotes TabHLH489 degradation. BR promotes wheat grain development by reducing *TabHLH489* expression through transcription factor TaBZR1. Natural variations in the promoter of *TabHLH489* in CS and SX results in the different expression of *TabHLH489* in CS and SX by affecting the binding ability of TaBZR1. Therefore, the TaSnRK1α1‐TabHLH489 regulatory module integrates BR and sugar signalling to regulate wheat grain size. Black bars indicate transcriptional repression. Red arrows and bars indicate posttranslational activating and inhibitory effects on protein. Green dashed lines indicate TabHLH489 produced by gene expression. Blue dashed lines show indirect mechanisms.

To investigate whether the SNP in P6 was selected during the breeding process in China, we further genotyped the Chinese wheat mini‐core collection (MCC), which consists of 262 accessions and represents much of the genetic diversity in the Chinese national collection. The percentage of accessions with *Hap‐S* was lower in introduced cultivars from other countries (21.05%) compared to landraces (41.96%) and modern cultivars (33.64%) (Figure 9a). Interestingly, the frequency of *Hap‐S* was observed to decline sharply during the early stages of the breeding process, from 50.00% in stage 1949–1957 to 19.64% in stage 1958–1978 and remained low in frequency, present in only 17.65% of cultivars post‐2000 (Figure 9b). Additionally, we observed distinct distribution characteristics of the alleles in the major Chinese agro‐ecological zones, with the zone III and VI exhibiting a low frequency of *Hap‐S* compared to other zones (Figure 9c). However, the grain length of varieties in these major Chinese agro‐ecological zones was increased accompanied by the frequency of *Hap‐S* (Figure 9d). Collectively, our findings indicate the potential selection of the short grain *Hap‐C* of *TabHLH489* during the breeding process in China.

## Discussion

Wheat grain weight is a complex quantitative trait and is largely dependent on grain size. In the present study, we demonstrated that the critical roles of TabHLH489 and TaSnRK1α1 in wheat grain development. Overexpressing *TaSnRK1α1* or knocking out *TabHLH489* both promotes wheat grain development and increases grain weight. Sugar induces the accumulation of TaSnRK1α1, which interacts with and phosphorylates TabHLH489 to facilitate its degradation. Additionally, we found that BR promotes wheat grain development through reducing *TabHLH489* expression through transcription factor TaBZR1. By integrating BR and sugar signals, the TaSnRK1α1‐TabHLH489 module regulates wheat grain development (Figure 9e).

### TabHLH489 acts as a negative regulator of wheat grain development

Grain weight is an important agronomic trait for cereal crops and several genes affecting wheat grain have been isolated through QTL mapping or homologous gene analysis. Here, a key locus *qGL2D* was identified as a regulator of grain length and grain weight through GWAS. This locus has been previously identified by different research groups studying varieties with different grain sizes (Cui *et al*., 2014; Ramya *et al*., 2010; Su *et al*., 2018; Xie *et al*., 2015). Our research further revealed that *TabHLH489* is a potential gene responsible for *qGL2D* locus. The correlation analysis and transgenic materials both showed lower expression levels of *TabHLH489* resulting increased grain length. Furthermore, the knockout of *TabHLH489* on chromosome 2D alone promoted grain length and additional knockouts on chromosome 2A and 2B can further enhance the grain length and regulate the downstream gene expression. These findings suggested that TabHLH489 negatively regulates wheat grain development in a dosage‐dependent manner.

The *qGL2D* locus consists of four genes, two of which are not expressed in wheat grains. However, *TabHLH489* and *TraesCS2D02G499500* exhibit the high expression levels in wheat grains and their expression levels are negatively correlated with wheat grain length. We have chosen to focus on studying *TabHLH489* rather than *TraesCS2D02G499500* due to the following two considerations. Firstly, the negative correlation between the expression level of *TraesCS2D02G499500* in seeds and wheat grain length was significantly lower compared to that of *TabHLH489*. Secondly, *TraesCS2D02G499500* encodes a homologue of Arabidopsis PELOTA, which is involved in RNA quality control systems during translation and plays an important role in immunity and salt stress responses (Ge *et al*., 2023; Kong *et al*., 2021). Conversely, *TabHLH489* encodes homologous genes of Arabidopsis, UPB1, AIF1‐AIF4 and IBH1, all of which are involved in inhibiting plant cell elongation (Bai *et al*., 2012; Wei and Chen, 2018; Zhang *et al*., 2009). However, the effect of *qGL2D* on grain length surpassed that of knocking out *TabHLH489*. It implies that *qGL2D* may be a compound locus in regulating grain length. Thus, the function of *TraesCS2D02G499500* in grain development needs to be studied in the future.

### TabHLH489 regulates wheat grain development downstream of the BR signalling pathway

Brassinosteroids are a class of growth‐promoting hormones and play pivotal roles in crop grain development. In rice, mutations that results in BR deficiency or BR insensitivity led to shorter and lighter grains (Cheng *et al*., 2020; Gao *et al*., 2019; Hong *et al*., 2003; Wu *et al*., 2016). This study confirms the critical role of BRs in wheat grain development. Enhancing BR signal by knocking out *TaSK2* or overexpressing *TaBZR1* led to the increased grain length and grain weight, while weakening the BR signal by knocking out *TaBRI1* or the overexpression of *TaSK2* resulted in the decreased grain length and grain weight. The expression of *TabHLH489* was decreased in the transgenic materials with the knockout of *TaSK2* or the overexpression of *TaBZR1*, but increased in the materials with the knockout of *TaBRI1* or the overexpression of *TaSK2*. Additionally, TaBZR1 directly binds to the promoter of *TabHLH489* to inhibit its expression. These findings suggest that TabHLH489 regulates wheat grain development downstream of BR signalling pathway.

Plant height and grain size are two crucial agronomic traits that greatly impact the crop yield. Excessively tall plants are prone to lodging, while excessively short plants tend to produce smaller grains, more ineffective tillers and exhibit weak disease resistance (Liu *et al*., 2018). Hence, achieving an optimal balance in plant height and grain size is vital for improving the crop yield. BR plays a significant role in regulating these traits in crops. Numerous BR‐related genes have been identified in rice, most of which influence both plant height and grain size. Mutants associated with BR synthesis in rice, such as *brd2*, *d11*, *d2* and *brd1*, exhibit varying degrees of reduced plant height and grain size (Hong *et al*., 2003, 2005; Mori *et al*., 2002; Tanabe *et al*., 2005). In our study, we uncovered a novel component of the BR signalling pathway called *TabHLH489*, which negatively regulates plant height and grain development. Overexpressing *TabHLH489* in wheat led to decreased plant height and grain size, while knocking out *TabHLH489* had no significant effects on plant height and resulted in increased grain weight. Additionally, both BR and *TabHLH489* were found to regulate leaf angle, which is pivotal for photosynthesis and plant density. Manipulating *TabHLH489* expression to reduce BR signalling resulted in more upright leaves. Therefore, targeting BR and TabHLH489 presents an excellent opportunity for wheat breeding. By modulating the activity of TabHLH489, the intensity of BR signal transduction can be adjusted, ultimately yielding wheat plants with semi‐dwarfed height, upright leaves and appropriately sized grains.

### TabHLH489 is a potential target for breeding practices

In breeding practices, grain shape and size are important factors in determining the production and market value of bread wheat (Gegas *et al*., 2010). The significant increase in grain width in wheat breeding has led to a shift from long and thin grains to larger, shorter and wider grains, primarily through modifications in grain length during domestication (Eckardt, 2010; Gegas *et al*., 2010; Yoshioka *et al*., 2019). Despite the trade‐off between number and size of grains, genetic gains in grain yield potential have been predominantly achieved by increasing grain number per spike, whilst selecting for stable or even reduced grain size (Jia *et al*., 2013; Shukla *et al*., 2015; Zhai *et al*., 2018). Analysis of each agro‐ecological zone revealed that grain length was negatively correlated with the grain number per spike (Figure S17a–c). However, the grain number per spike has no remarkable correlation with these two haplotypes (Figure S17b,c).The widespread use of the short‐grain *Hap‐C* of *TabHLH489* in past Chinese breeding processes may be an indirect outcome of the balance between selecting for shorter but wider grains and the number versus size of grains. Thus, the short‐grain *Hap‐C* of TabHLH489 allele may be crucial in achieving larger grains with minimal trade‐off in terms of grain number per spike, although there is still room for improvement, particularly in grain length in zones III and VI.

In conclusion, TabHLH489 plays a crucial role in wheat grain development, acting downstream of the BR signalling pathway. TaSnRK1α1 interacted with and phosphorylated TabHLH489 to induce its degradation, thereby promoting wheat grain development. Natural variation in the proximal (P6) region of *TabHLH489* promoter contributes to the expression difference between the two haplotypes of *TabHLH489* by affecting TaBZR1 binding capacity. However, the superior haplotype within the proximal (P6) region of the *TabHLH489* promoter did not emerge prominently during domestication and breeding practices. These findings suggest that further investigation is needed to uncover the underlying mechanisms of *TabHLH489* in regulating wheat development and grain yield, with the aim of potentially reintegrating the superior haplotype into wheat breeding.

## Materials and methods

### Phenotyping and GWAS

A total of 364 wheat accessions were evaluated in six different environments using a plant density of 2.7 million ha^−1^. Field trials were conducted at Shijiazhuang (37.85° N, 114.82° E) and Dezhou (37.43° N, 116.35° E) in China over three consecutive cropping seasons (2015–2018), with two replicates carried out in a randomized complete block design. Each block consisted of six rows, each three meters in length and spaced 21 cm apart. Agronomic management followed local cultivation practices and no serious damage from plant diseases, insect pests, or lodging occurred during the growing seasons. A total of 30 representational main spikes from the inner rows were harvested and used to measure grain shape and size. Grain weight was determined with three replicates, using more than 200 random grains for each replicate and then transformed into 1000‐grain weight (TGW, g). The respective average values for grain length (GL, mm), grain width (GW, mm), grain length/width (GLW), grain area (GA, mm^2^) and grain circumference (GC, mm) were calculated using a Crop Grain Appearance Quality Scanning Machine (SC‐G, Hangzhou Wanshen Technology Co., Ltd., China). Each accession was genotyped using Affymetrix Wheat660K SNP arrays by Capital Bio Corporation (Beijing, China). SNPs were filtered based on the following criteria to ensure high quality SNP markers: (1) Minor allele frequency (MAF) not less than 5%; (2) Missing rate in the population does not exceed 10%; (3) Genotype hybrid rate less than 5%; and (4) Unique mapping to the reference genome IWGSC RefSeq V1.0. High‐quality SNPs from 364 samples were analysed for association with phenotypic data using Tassel v5.2 using the mixed linear model (introducing PCA as a fixed effect and Kship matrix as a random effect in the model). The independent effective markers (Ne) were estimated based on linkage disequilibrium using GEC software (Li *et al*., 2012). The *P* value of 1/Ne was used as the suggestive threshold for declaring a significant association, in accordance with the adjusted Bonferroni method. Manhattan plots and quantile‐quantile plots were generated using R package ‘CMplot’ (https://github.com/YinLiLin/R‐CMplot). Pairwise *R* ^2^ values were calculated and displayed with LD plots using Haploview 4.2 software (Barrett *et al*., 2005).

### Map‐based cloning of *TabHLH489*

SX is a commercial variety known for its increased grain length and grain weight, while CS is a landrace with short grain length and low grain weight. To develop F_1_ hybrids, SX was used as the male parent and CS as the female parent. The SSR marker Xwmc181.2 and InDel marker IN507, which were adjacent to the locus *qGL2D*, were used as assistant markers for genetic population construction due to their polymorphism between two parental lines. The BC_3_F_3_ population for fine mapping of *TabHLH489* and near‐isogenic lines (NIL^*qGL2D*^) were generated by three or five generations of backcrossing CS × SX F_1_ progeny to CS. The resulting BC_3_F_2_ plants were self‐pollinated to produce BC_3_F_3_ progenies, which were used to perform fine mapping analysis of *TabHLH489*. The BC_3_F_3_ populations were grown at Yuanxia village, Yantai, Shandong, China (37.53° N, 121.15° E), with spacing of 0.4 meters between rows and 0.2 meters between individual plants during the 2021–2022 growing season. To isolated the recombinant lines, DNAs were extracted from the immature leaves of the 9368 individual plants in the BC_3_F_3_ populations using the CTAB method. PCR genotyping was conducted by using markers in the *qGL2D* region. Markers' information was shown in Table S2. The progenies of recombinants were used to test the grain length trait.

### Vector construction and wheat transformation

The full‐length CDS without stop codon of *TabHLH489*, *TaSK2*, *TaBZR1* and *TaSnRK1α1* were amplified from cDNA of CS, while *TabHLH489‐S* was amplified from cDNA of SX, using specific primers. The promoters of *TabHLH489* and *TabHLH489‐S* were cloned from genomic DNA of CS and SX respectively, using their own primers. All PCR products were cloned into pENTR™/SD/D‐TOPO™ vector (Thermo Scientific, USA) and then recombined with the vectors, including pLGY02 (*pUbi:C‐GFP*), pBI221 (*p35S:C‐GFP*), pGreenII (*p35S:C‐Luciferase*), pDEST15 (N‐GST), pMAL2CGW (N‐MBP), pX‐YFP (*p35S:C‐YFP*) and p1390 (*p35S:C‐Myc*). To generate gene knockout mutants, the sgRNAs were designed based on their targets on the website CRISPRdirect (http://crispr.dbcls.jp/). The expression cassettes containing sgRNAs driven by *TaU3* promoter were introduced into the binary vector pBUE411 using BsaI and T4 ligase (New England Biolabs, USA), to generate the binary vectors for targeting *TabHLH489‐2A/2B/2D*, *TaSK2‐1A/1B/1D*, *TaBRI1‐3A/3B/3D* and *TaSnRK1α1‐1A/1B/1D*, respectively (Xing *et al*., 2014). Primer pairs used for vectors construction were listed in Table S2. All binary vector constructs were introduced into *Agrobacterium tumefaciens* (strain EHA105) and transformed into Fielder plants as previously described (Zhang *et al*., 2018).

The wheat wild type and all transformation backgrounds used in this study were the Fielder variety. Transgenic plants and near‐isogenic lines for phenotype analysis were planted in a greenhouse with a 16‐h light (22 °C)/8‐h dark (16 °C) cycle and approximately 50% relative humidity.

### Histological assay

Grains from three individual plants at 15‐DPA stage were sampled and fixed in Carnoy's fluid (absolute alcohol: acetic acid = 3:1, v/v) at room temperature for 24 h. After fixation, grains were dehydrated in 100%, 90%, 80% and 70% ethanol for 1 h sequentially and stored in 70% ethanol at last. Pericarp pieces of 1–2 mm^2^ were taken from the middle of grains and stained using a modified Pseudo‐Schiff Propidium Iodide (PS‐PI) method (Truernit *et al*., 2008). Cell lengths of pericarps were pictured by the LSM‐900 laser scanning confocal microscope (Zeiss, Germany) and measured by ImageJ 1.51 (http://imagej.net/ij/).

### Ratiometric bimolecular fluorescence complementation (rBiFC) assay

For the bimolecular fluorescence complementation assays of TaSnRK1α1 and TabHLH489, the full‐length CDS of *TaSnRK1α1* and *TabHLH489* were cloned into pDONR221‐P1P4 and pDONR221‐P3P2 using the BP recombination reaction (Invitrogen, USA), respectively. The sequencing‐confirmed pDONR221 clones were recombined with pBiFCt‐2in1‐NN to generate the vector *p35S:nYFP‐TabHLH489‐p35S:cYFP‐TaSnRK1α1‐p35S:RFP* and *p35S:nYFP‐H3‐p35S:cYFP‐TaSnRK1α1‐p35S:RFP* (Negative control) (Hecker *et al*., 2015). The resulting vectors were transformed into mesophyll protoplasts to detect the protein interaction between TaSnRK1α1 and TabHLH489 as previously described (Yoo *et al*., 2007). Fluorescent signals were observed using the LSM‐900 laser scanning confocal microscope (Zeiss).

### Pull‐down assay

The recombinant GST‐fused TaSnRK1α1 and MBP‐fused TabHLH489 were extracted from bacteria using glutathione beads (GE Healthcare, USA) and amylose resin (New England Biolabs), respectively. To validate the protein interaction, GST‐TaSnRK1α1 (1 μg) was incubated with MBP‐TabHLH489 (1 μg) or MBP (1 μg) at 4 °C for 1 h and the beads were washed six times with wash buffer. The proteins were eluted from glutathione beads by boiling in 50 μL 2 × SDS loading buffer and separated by an 8% SDS‐PAGE gel. Gel blots were analysed using anti‐MBP (New England Biolabs, 1:5000 dilution) and anti‐GST antibodies (Merck, 1:3000 dilution, Germany).

### Co‐immunoprecipitation (CoIP) assay

The recombinant binary vectors *p35S:TaSnRK1α1‐Myc* and *p35S:TabHLH489‐YFP* were transformed into *Agrobacterium tumefaciens* strain GV3101. The *Agrobacterium* suspensions containing *p35S:TaSnRK1α1‐Myc* or both *p35S:TaSnRK1α1‐Myc* and *p35S:TabHLH489‐YFP* were injected into the leaves of *Nicotiana benthamiana*, respectively. After infiltration, the plants were kept in the greenhouse at 22 °C for 48 h. To conduct immunoprecipitation assay, total plant proteins were extracted from 1 g leaves (without main vain) using plant extraction buffer (50 mM Tris–HCl pH 7.5, 150 mM NaCl, 1 mM EDTA, 10% Glycerol, 0.05% Tween‐20, 10 mM NaF, 2 mM NaVO_4_, 25 mM β‐Glycerophosphate disodium, 1 mM PMSF and 1 × protease inhibitor). GFP‐trap beads were then added into the protein extraction and incubated on a rotator at 4 °C for 2 h. The beads were washed with plant extraction buffer containing 0.15% NP‐40 for 3 times. The proteins were eluted from GFP‐trap beads by boiling in 60 μL 2 × SDS loading buffer and separated by 8% SDS‐PAGE gels. Gel blots were analysed by anti‐GFP (TransGen, 1:5000 dilution, China) and anti‐Myc (Sigma, 1:5000 dilution, USA) antibodies.

### Immunoblot assay

Total proteins were extracted from 7‐day‐old seedlings of Fielder, *TaSnRK1α1‐Ox*, *TabHLH489‐Ox* and crossing line *TaSnRK1α1‐Ox/TabHLH489‐Ox* using plant extraction buffer (as mentioned above) with 0.05% Tween‐20, 10 mM NaF, 2 mM NaVO4, 25 mM β‐Glycerophosphate disodium, 1 mM PMSF and 1 × protease inhibitor. The amounts of total proteins were quantified using Bradford solution on the Infinite M200 Pro microplate spectrophotometer (Tecan, Switzerland). Then 120 μg total proteins were separated by 8% SDS‐PAGE after boiling with proper volume of 5 × SDS loading buffer and transferred to PVDF membranes, blocked with 5% (w/v) fat‐free milk in TBST buffer (20 mM Tris–HCl pH 7.5, 150 mM NaCl and 0.1% Tween‐20 (v/v) for 1 h). The membranes were detected with anti‐GFP (TransGen, 1:5000 dilution) or anti‐Actin (Sigma, 1:5000 dilution) antibodies and subsequently incubated with the secondary antibody anti‐mouse‐HRP (Bio‐Rad, 1:5000 dilution, USA). The TaActin protein was used as a loading control.

The stability assays for both TaSnRK1α1 and TabHLH489 proteins were carried out on 7‐day‐old seedlings of *OE‐TaSnRK1α1* and *OE‐TabHLH489* plants after a 6‐h treatment with 1% sucrose under light conditions. The total protein extraction and GFP‐tagged protein abundance were performed as mentioned above.

### Cell‐free degradation assay

Total proteins were extracted from 7‐day‐old seedlings of Fielder and *OE‐TaSnRK1α1* using the modified cell‐free degradation buffer (25 mM Tris–HCl, pH 7.5, 10 mM NaCl, 10 mM MgCl_2_, 1 mM PMSF, 5 mM DTT and 2 mM ATP) as previously described (Wang *et al*., 2009). The extracted protein concentrations from Fielder and *OE‐TaSnRK1α1* were adjusted to the equal concentration of 5 μg/μL in the degradation buffer. The recombinant proteins MBP‐TabHLH489 and MBP were purified from *Escherichia coli* and incubated in 100 μL total protein extracts from Fielder or *OE‐TaSnRK1α1* for 5 min, 10 min and 15 min at 22 °C, respectively. Samples were taken from each reaction at the indicated time points. MBP‐TabHLH489 and MBP abundance were determined by immunoblot analysis as mentioned above and the degradation ratio of MBP‐TabHLH489 and MBP in Filder and *OE‐TaSnRK1α1* were calculated as the ratios between MBP‐TabHLH489 and TaRubisco, which was used as a loading control and stained with CBB R250. The protein abundance of each time point was quantified by ImageJ 1.51.

### Chromatin immunoprecipitation (ChIP)‐qPCR assay

The young leaves from 7‐day‐old Fielder and *OE‐TaBZR1* plants were harvested and crosslinked with 1.0% formaldehyde (v/v) for 30 min by vacuum infiltration. The crosslinking reaction was stopped by replacing the formaldehyde solution with 0.25 M glycine. The anti‐GFP polyclonal antibody (ab290, Abcam, England) was incubated with Protein A/G Magnetic Beads at 4 °C for 2 h on a rotator. For immunoprecipitation, the sonicated chromatin extracts were combined with the antigen–antibody‐beads complex and incubated at 4 °C for 2 h. The beads were washed sequentially with low salt wash buffer, high salt wash buffer, LiCl buffer and TE buffer, as previously described (Tian *et al*., 2018). The protein‐DNA complex was eluted with the elution buffer (0.1 M NaHCO_3_ and 1% SDS, w/v) and de‐crosslinked with 0.2 M NaCl at 65 °C overnight. Proteins were removed using proteinase K (Thermo Scientific) and DNA was precipitated using 3 volumes of ethanol and 1/10 volume of 3 M sodium acetate. The fold enrichment of the promoter of *TabHLH489* was calculated as the ratio between Fielder and *OE‐TaBZR1*. The promoter of *TaADPRF* was used as an internal control. The ChIP‐qPCR experiments were carried out with three independent biological repeats. Primer pairs used for ChIP‐qPCR analysis were listed in Table S2.

### DNA‐protein pull‐down assay

The recombinant protein MBP‐TaBZR1 was purified from *Escherichia coli*. using amylose resin. Fragments P6‐CS and P6‐SX were amplified by PCR using 5′‐end biotin‐labelled primers (Table S2). The fragments were incubated with MBP‐TaBZR1 in the 1 × binding buffer [100 mM HEPES (pH 7.9), 500 mM KCl, 25 mM NaCl, 1 mM EDTA, 25% glycerol (v/v), 0.5% TritonX‐100 (v/v), 1 ng·μL^−1^ poly dI‐dC] at 22 °C for 45 min and pulled down using streptavidin‐agarose beads. The abundance of MBP‐TaBZR1 abundance on the promoter of *TabHLH489* was calculated as the ratio between pulled down MBP‐TaBZR1 and input MBP‐TaBZR1 using ImageJ 1.51.

### RNA‐seq

For RNA‐Seq analysis, 5‐DPA seeds from Fielder plants and *OE‐TabHLH489* plants grown in the greenhouse were collected to isolate the total mRNA and construct the mRNA sequencing libraries. Sequencing was performed on the BGISEQ‐500 platform at Beijing Genomics Institute. The RNA‐seq clean reads were mapped to the Chinese Spring reference genome (International Wheat Genome Sequencing Consortium RefSeq v1.1) downloaded from Ensembl Plants using Bowtie2 software. Htseq‐count with default parameters was used to calculate the gene read counts, which were then normalized to TPM (**T**ranscripts **P**er Kilobase per **M**illion mapped reads) and calculated the gene relative expression level by Salmon. Differential gene expression analysis was carried out using DESeq2 with genes showing log_2_(fold change) ≥ 0.5849 and adjusted *P* value (FDR) < 0.05 were considered to be differentially expressed ones. Enriched GO analysis of TabHLH489‐regulated genes was identified using Cluster Profiler.

## Conflict of interest

The authors declare that there are no competing interests.

## Author contributions

J.L., D.W., J.X., M.F. and M.‐Y.B. together designed the experiments. J.L. and N.S. performed statistical analysi of plant growth, transient expression assay, pull‐down assay, kinase assay, western blot and RT‐qPCR. D.W. and J.L. performed GWAS and QTL mapping analysis. D.W. carried out natural variations analysis. J.L., N.S., F.Y., X.L. generated *OE‐TaSnRK1α1* and *tasnrk1α1‐ko* plants. J.L. and F.Y. generated *tabri1* mutants. J.M., R.Z., G.Z., X.Y. and C.Z. participated the phenotypic identification. J.L. performed all other experiments. C.H., M.F., G.X. and G.L. provided critical discussion on the work. J.L., D.W., J.X., M.F. and M.‐Y.B. wrote the manuscript.

## Supporting information


**Figure S1** GWAS and candidate gene characterization for wheat grain length. (a) Genome wide association study for wheat grain length. The red horizontal dashed line indicates the significance threshold *P* value (*P* = 1.0E−04) for marker‐trait associations. (b) Quantile‐quantile plot for the GWAS under a mixed linear model. (c) The expression levels of four genes in 5‐DPA seeds of CS and SX. ‘***’ and ‘ns’ indicates statistically significant differences and no significant differences between samples (Students's *t*‐test, *P* < 0.001), respectively. (d) Association analysis of *TraesCS2D02G499500* expression level and grain length in 10‐DPA seeds of 102 representative wheat varieties.

**Figure S2** Characterization of *TabHLH489*. (a) Spatio‐temporal expression pattern of *TabHLH489* in CS. Error bars indicate ±SD (*n* = 3). *TaADPRF* was used as an internal control. (b) Subcellular localization of *TabHLH489* in tobacco. Scale bar = 50 μm.

**Figure S3** CRISPR/Cas9‐mediated mutations at *TabHLH489* target sites in plants. (a) Schematic illustration of the target sites. The blue box indicates the exon of the *TabHLH489* gene. The red box indicates the sgRNAs targeting sites. (b) The mutation sites of *TabHLH489* are indicated in red. The underlined nucleotides represent PAM (protospacer‐adjacent motif) sequences.

**Figure S4** Knockout of *TabHLH489* on Chr2D promotes wheat grain length. (a) Wheat grain morphology of *TabHLH489* single mutant (*AABBdd*). Scale bars = 1 cm. (b–d) Thousand grain weight, grain length and grain width of *TabHLH489* single mutant compared to Fielder. The grains (*n* > 300) came from six individual plants on average. ‘*’ and ‘***’ indicates statistically significant differences between samples (Student's *t*‐test, *P* < 0.05 and *P* < 0.001, respectively).

**Figure S5** Protein alignment analysis of TabHLH489 sequence from CS and SX. TabHLH489 and TabHLH489‐S indicate proteins from wheat CS and SX, respectively. Green background colour indicates sequence variance between two proteins.

**Figure S6** The overexpression of *TabHLH489* from SX resulted in the decreased grain length and grain weight. (a) Quantitative RT‐PCR analysis of the expression level of *TabHLH489* in 5‐DPA seeds of *OE‐TabHLH489* and *OE‐TabHLH489‐S* overexpression plants. Error bars indicate ±SD from three biological repeats. *TaADPRF* was used as an internal control. (b, c) Plant architecture of Fielder and *OE‐TabHLH489‐S* plants at the heading stage. Error bars indicate ±SD (*n* ≥ 6). Scale bar = 10 cm. (d–f) Wheat grain morphology of Fielder and *OE‐TabHLH489‐S* lines. The grains (*n* > 100) came from six individual plants on average. Scale bar = 1 cm. (g) Quantitative RT‐PCR analysis of *TaEXPA2* and *TaEXPA4* in 5‐DPA seeds of Fielder, *OE‐TabHLH489‐S* plants. Error bars indicate ±SD (*n* = 3). *TaADPRF* was used as an internal control. (h, i) The grain pericarp cell length of Fielder and *OE‐TabHLH489‐S* lines. The cells (*n* > 100) came from six individual plants on average. Scale bar = 50 μm. (j, k) The flag leaf angles of Fielder and *OE‐TabHLH489‐S* lines. Error bars indicate ±SE, Scale bar = 5 cm. Different letters above bars indicate statistically significant differences between samples (one‐way ANOVA, *P* < 0.05). ‘***’ indicates statistically significant differences between samples (Student's *t*‐test, *P* < 0.001).

**Figure S7** Sequence variations between the promoters of *TabHLH489* from CS and SX. *pTabHLH489* and *pTabHLH489‐S* indicate promoters from wheat CS and SX, respectively. Green background colour indicates the sequence variance between two promoters.

**Figure S8** The promoter activity of *pTabHLH489* and *pTabHLH489‐S*. LUC activity was normalized to REN. Error bars indicate ±SD (*n* = 3). ‘*’ indicate statistically significant differences between two samples (Student's *t*‐test, *P* < 0.05).

**Figure S9** TaSnRK1α1 promotes wheat grain development. (a) Quantitative RT‐PCR analysis of the expression level of *TaSnRK1α1* in 5‐DPA seeds of Fielder and *OE‐TaSnRK1α1* plants. Error bars indicate ±SD from three biological repeats. *TaADPRF* was used as an internal control. (b, c) Plant architecture of Fielder and *OE‐TaSnRK1α1* plants at the heading stage. Error bars indicate ±SD (*n* ≥ 6). Scale bar = 10 cm. (d) The mutation sites of *tasnrk1α1‐ko* mutants are indicated in red. (e, f) Plant architecture of Fielder and *tasnrk1α1‐ko* mutants at the heading stage. Error bars indicate ±SD (*n* ≥ 6). Scale bar = 10 cm. (g, h) Wheat grain morphology of Fielder and *tasnrk1α1‐ko* mutants. The grains (*n* > 300) came from six individual plants on average. Scale bar = 1 cm. (i, j) The grain pericarp cell length of Fielder and *tasnrk1α1‐ko* mutants. The cells (*n* > 100) came from six individual plants on average. Scale bar = 50 μm. Different letters above bars indicate statistically significant differences between samples (one‐way ANOVA, *P* < 0.05).

**Figure S10** TaSnRK1α1 inhibits activity of TabHLH489 in the crossing progenies. (a) Quantitative RT‐PCR analysis of the expression level of *TabHLH489* in 5‐DPA seeds of Fielder, *OE‐TaSnRK1α1*, *OE‐TabHLH489* and *OE‐TaSnRK1α1/OE‐TabHLH489* plants, respectively. Error bars indicate ±SD from three biological repeats. *TaADPRF* was used as an internal control. (b, c) The grain pericarp cell length of Fielder, *OE‐TabHLH489*, *OE‐TaSnRK1α1* and *OE‐TaSnRK1α1/OE‐TabHLH489* plants. The cells (*n* > 100) came from six individual plants on average. Scale bar = 50 μm. Different letters above bars indicate statistically significant differences between samples (one‐way ANOVA, *P* < 0.05).

**Figure S11** Quantitative RT‐PCR analysis of BR effects on *TabHLH489* expression. Total RNA was extracted from the leaves of 7‐day‐old seedlings which were foliar‐sprayed with 100 nM eBL (containing 0.01% TritonX‐100) for different time. Error bars indicate ±SD (*n* = 3). *TaADPRF* was used as an internal control. Different letters above bars indicate statistically significant differences between samples (one‐way ANOVA, *P* < 0.05).

**Figure S12** *TaBRI1* knocked out affects wheat grain development. (a) The mutation sites of *TaBRI1* knockout mutants are indicated in red. (b, c) Plant architecture of Fielder and *tabri1* mutants at the heading stage. Error bars indicate ±SD (*n* ≥ 6). Scale bar = 10 cm. (d, e) The flag leaf angles of Fielder and *tabri1* mutants. Scale bar = 5 cm. Error bars indicate ±SE of different lines. (f–h) Wheat grain morphology of Fielder and *tabri1* mutants. The grains (*n* > 300) came from six individual plants on average. Scale bar = 1 cm. (i, j) The grain pericarp cell length of Fielder and *tabri1* mutants. The cells (*n* > 100) came from six individual plants on average. Scale bar = 50 μm. Different letters above bars indicate statistically significant differences between samples (one‐way ANOVA, *P* < 0.05).

**Figure S13** *TaSK2* inhibited wheat grain development. (a) The mutation sites of *TaSK2* mutant are indicated in red. (b) Quantitative RT‐PCR analysis of the expression level of *TaSK2* in 5‐DPA seeds of *OE‐TaSK2* plants. Error bars indicate ±SD from three biological repeats. *TaADPRF* was used as an internal control. (c, d) Plant architecture of Fielder, *OE‐TaSK2* and *task2‐aabbdd* mutant at the heading stage. Error bars indicate ±SD (*n* ≥ 6). Scale bar = 10 cm. (e, f) The flag leaf angles of Fielder, *OE‐TaSK2* and *task2‐aabbdd* mutant. Scale bar = 5 cm. Error bars indicate ±SE. (g–i) Wheat grain morphology of Fielder, *OE‐TaSK2* and *task2‐aabbdd* mutant. The grains (*n* > 300) came from six individual plants on average. Scale bar = 1 cm. (j, k) The grain pericarp cell length of Fielder, *OE‐TaSK2* and *task2‐aabbdd* mutant. The cells (*n* > 100) came from six individual plants on average. Scale bar = 50 μm. Different letters above bars indicate statistically significant differences between samples (one‐way ANOVA, *P* < 0.05).

**Figure S14** *TaBZR1* promotes wheat grain development. (a) Quantitative RT‐PCR analysis of the expression level of *TaBZR1* in 5‐DPA seeds of Fielder and *OE‐TaBZR1* plants. Error bars indicate ±SD from three biological repeats. *TaADPRF* was used as an internal control. (b, c) Plant architecture of Fielder and *OE‐TaBZR1* plants at the heading stage. Error bars indicate ±SD (*n* ≥ 6). Scale bar = 10 cm. (d, e) The flag leaf angles of Fielder and *OE‐TaBZR1* plants. Error bars indicate ±SE, Scale bar = 5 cm. (f–h) Wheat grain morphology of Fielder and *OE‐TaBZR1* plants. The grains (*n* > 300) came from six individual plants on average. Scale bar = 1 cm. (i, j) The grain pericarp cell length of Fielder and *OE‐TaBZR1* plants. The cells (*n* > 100) came from six individual plants on average. Scale bar = 100 μm. (k) Quantitative RT‐PCR analysis of *TaEXPA2* and *TaEXPA4* in 5‐DPA seeds of Fielder and *OE‐TaBZR1* plants. Error bars indicate ±SD (*n* = 3). *TaADPRF* was used as an internal control. Different letters above bars indicate statistically significant differences between samples (one‐way ANOVA, *P* < 0.05). ‘**’ and ‘***’ indicates statistically significant differences between samples (Student's *t*‐test, *P* < 0.01 and *P* < 0.01, respectively).

**Figure S15** *TabHLH489* regulates the expression level of BR‐related genes in a dose‐dependent manner. (a) Quantitative RT‐PCR analysis of *TaBRI1* and *TaSK2* in 7‐day seedlings of Fielder and *TabHLH489* single, double and triple mutants. (b) Quantitative RT‐PCR analysis of *TaEXPA2* and *TaEXPA4* in 7‐day seedlings of Fielder and *TabHLH489* single, double and triple mutants. Error bars indicate ±SD (*n* = 3). *TaADPRF* was used as an internal control. Different letters above bars indicate statistically significant differences between samples (two‐way ANOVA, *P* < 0.05).

**Figure S16** Natural variations of the *TabHLH489* promoter region alter the binding site of TaBZR1. Green background colour indicates sequence variance. Red Letters indicate the core element of the binding site of TaBZR1.

**Figure S17** *TabHLH489* contributes a limitation of the trade‐off between grain number per spike and 1000‐grain weight. (a) The correlation analysis between the grain length and grain number per spike in wheat varieties from the major Chinese agro‐ecological zones. (b) The correlation analysis between the expression level of *TabHLH489* and grain number per spike in 102 representative varieties. (c) Grain number per spike of wheat varieties containing *Hap‐S* or *Hap‐C*.

**Table S1** DEGs in seeds of Fielder and *OE‐TabHLH489* at the 5‐DPA stage.

**Table S2** Information of primers used in this study.

## Data Availability

RNA‐seq data that support the findings of this study have been deposited at the National Center for Biotechnology Information Gene Expression Omnibus (GEO) and are accessible through the GEO series accession number GSE253343. Sequence data of genes referred to in this article can be found in the Ensembl Plants data library, including *TabHLH489* (*TraesCS2D02G499200*), *TabHLH489‐2A* (*TraesCS2A02G499000*), *TabHLH489‐2B* (*TraesCS2B02G527100*), *TaSK2* (*TraesCS1B02G142300*), *TaSK2‐1A* (*TraesCS1A02G125000*), *TaSK2‐1D* (*TraesCS1D02G129000*), *TaBZR1* (*TraesCS2A02G187800*), *TaBRI1‐3A* (*TraesCS3A02G245000*), *TaBRI1‐3B* (*TraesCS3B02G275000*), *TaBRI1‐3D* (*TraesCS3D02G246500*), *TaSnRK1α1* (*TraesCS1D02G353300*), *TaSnRK1α1‐1A* (*TraesCS1A02G350500*), *TaSnRK1α1‐1B* (*TraesCS1B02G364800*), *TaADPRF* (*TraesCS3A02G337300*), *TaD2* (*TraesCS3D02G106100*), *TaCPD* (*TraesCS5B02G133400*), *TaEXPA2* (*TraesCS3A02G344800*), *TaEXPA4* (*TraesCS1D02G299700*), *TaActin* (*TraesCS5B02G124100*) and *SnAK2* (*AT3G45240*).
