# Supplementary figures and images for: The TaSnRK1‐TabHLH489 module integrates brassinosteroid and sugar signalling to regulate the grain length in bread wheat
Source: Plant Biotechnol J. 2024 Feb 27;22(7):1989–2006. doi: 10.1111/pbi.14319 (PMC11182588; doi:10.1111/pbi.14319)

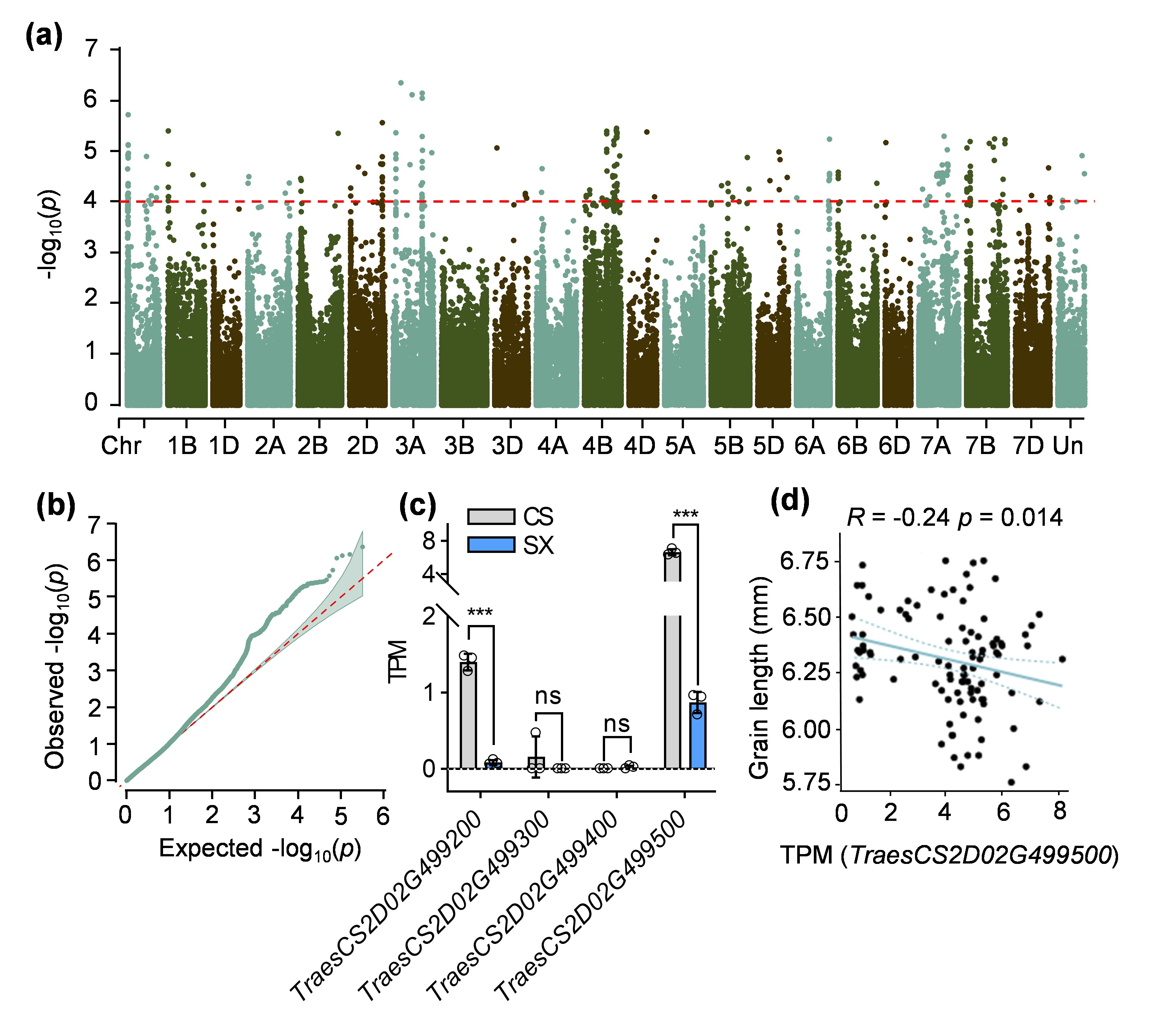

Supplement: Supplementary file 1 — Figure S1 GWAS and candidate gene characterization for wheat grain length. (a) Genome wide association study for wheat grain length. The red horizontal dashed line indicates the significance threshold P value (P = 1.0E−04) for marker‐trait associations. (b) Quantile‐quantile plot for the GWAS under a mixed linear model. (c) The expression levels of four genes in 5‐DPA seeds of CS and SX. ‘***’ and ‘ns’ indicates statistically significant differences and no significant differences between samples (Students's t‐test, P < 0.001), respectively. (d) Association analysis of TraesCS2D02G499500 expression level and grain length in 10‐DPA seeds of 102 representative wheat varieties. [file PBI-22-1989-s016.jpg]

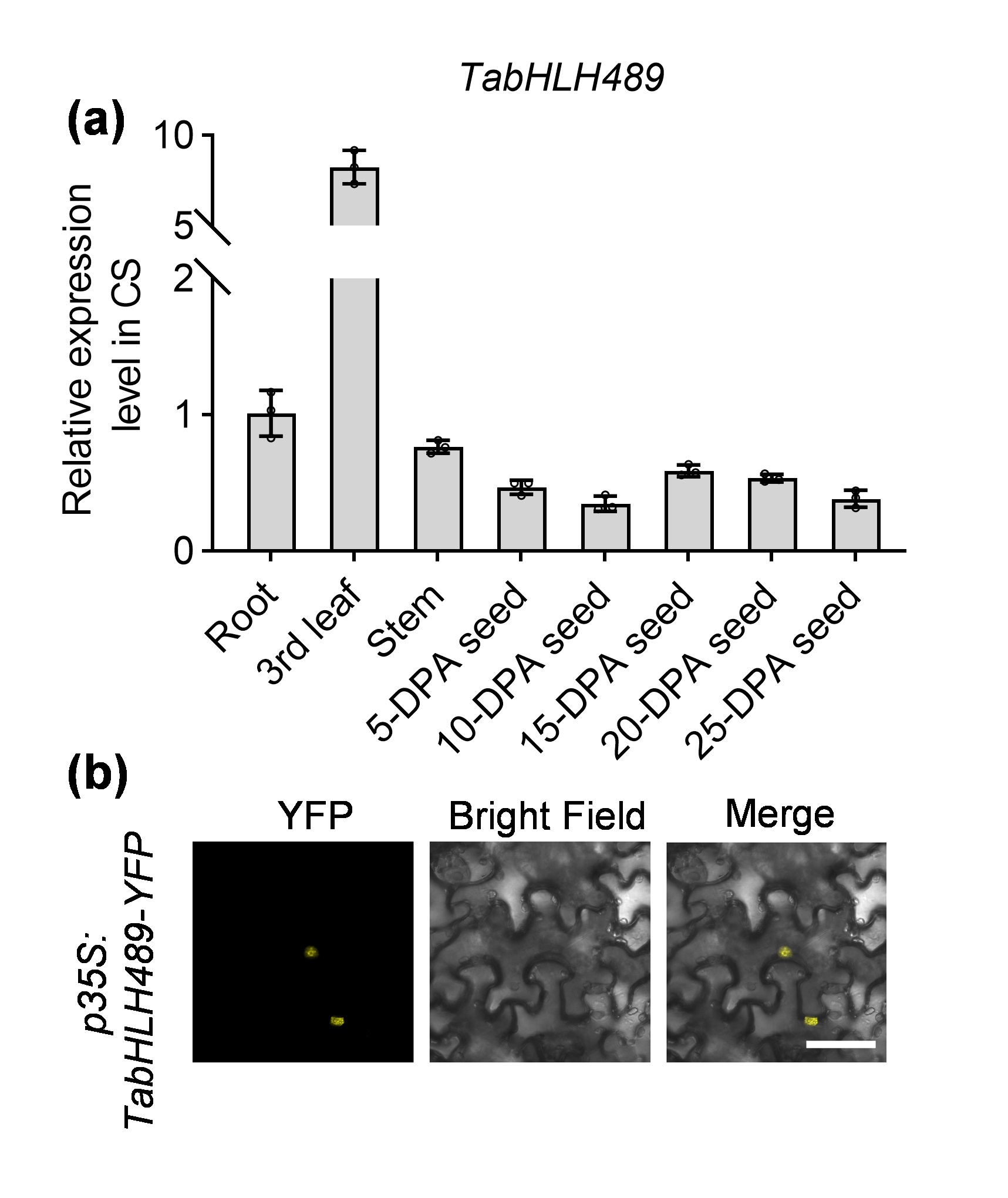

Supplement: Supplementary file 2 — Figure S2 Characterization of TabHLH489. (a) Spatio‐temporal expression pattern of TabHLH489 in CS. Error bars indicate ±SD (n = 3). TaADPRF was used as an internal control. (b) Subcellular localization of TabHLH489 in tobacco. Scale bar = 50 μm. [file PBI-22-1989-s007.jpg]

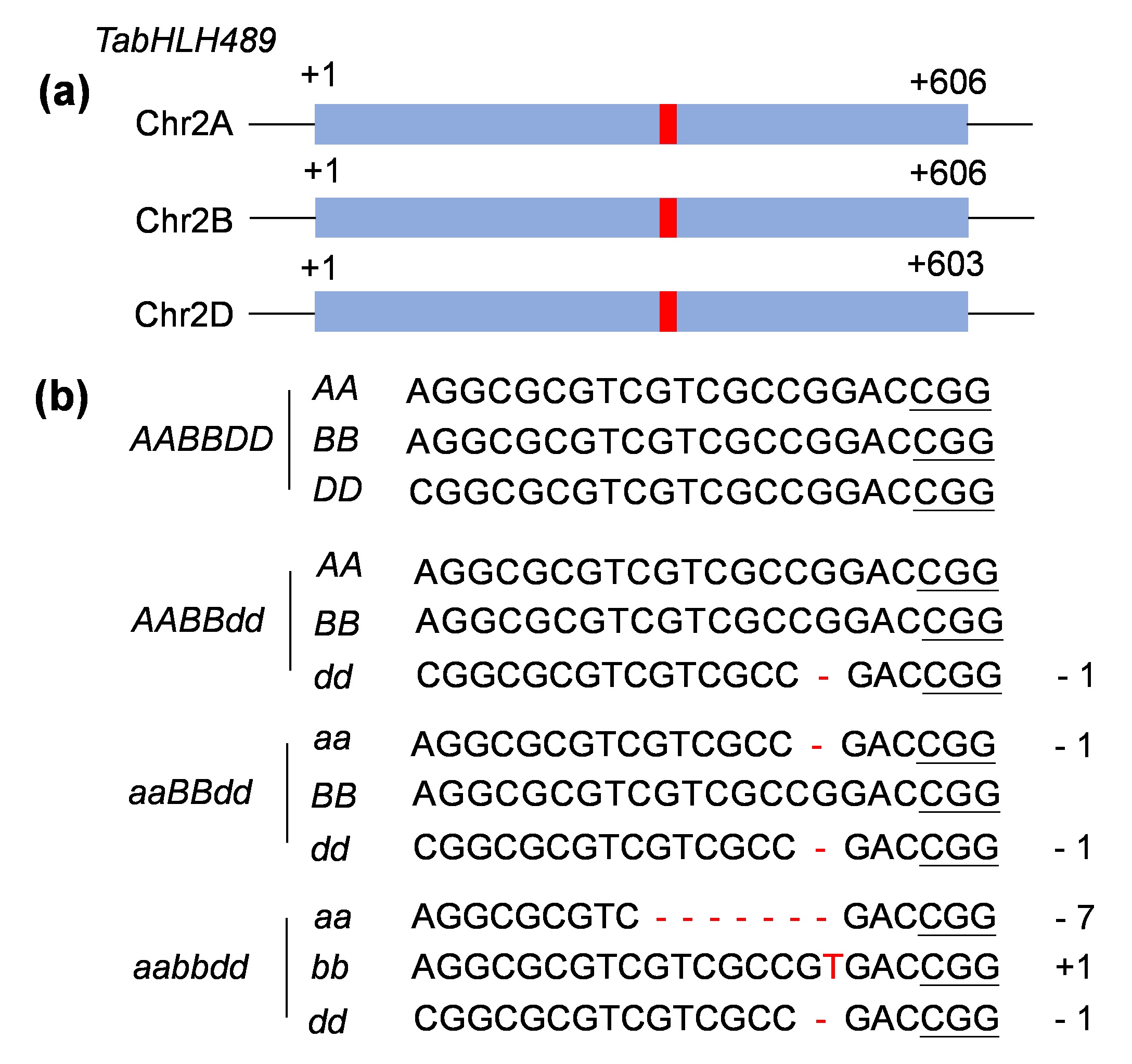

Supplement: Supplementary file 3 — Figure S3 CRISPR/Cas9‐mediated mutations at TabHLH489 target sites in plants. (a) Schematic illustration of the target sites. The blue box indicates the exon of the TabHLH489 gene. The red box indicates the sgRNAs targeting sites. (b) The mutation sites of TabHLH489 are indicated in red. The underlined nucleotides represent PAM (protospacer‐adjacent motif) sequences. [file PBI-22-1989-s013.jpg]

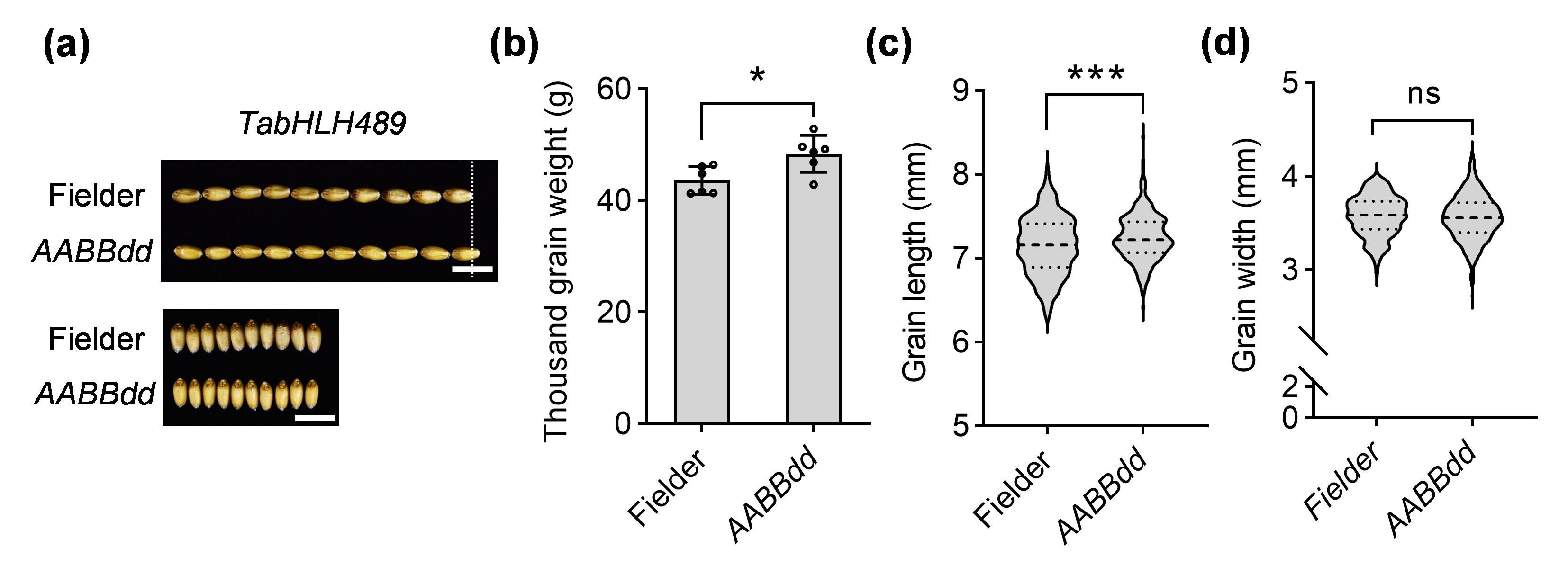

Supplement: Supplementary file 4 — Figure S4 Knockout of TabHLH489 on Chr2D promotes wheat grain length. (a) Wheat grain morphology of TabHLH489 single mutant (AABBdd). Scale bars = 1 cm. (b–d) Thousand grain weight, grain length and grain width of TabHLH489 single mutant compared to Fielder. The grains (n > 300) came from six individual plants on average. ‘*’ and ‘***’ indicates statistically significant differences between samples (Student's t‐test, P < 0.05 and P < 0.001, respectively). [file PBI-22-1989-s009.jpg]

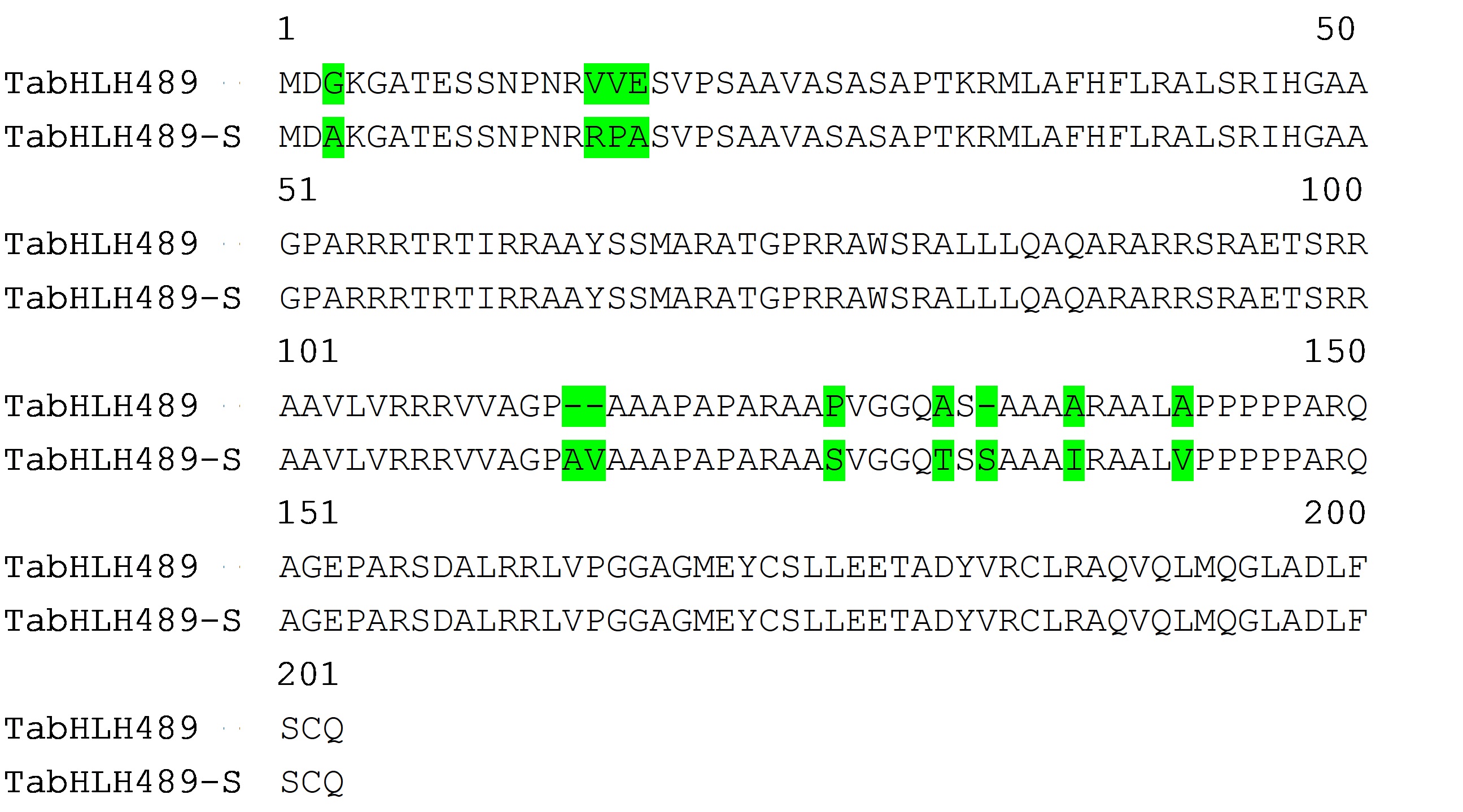

Supplement: Supplementary file 5 — Figure S5 Protein alignment analysis of TabHLH489 sequence from CS and SX. TabHLH489 and TabHLH489‐S indicate proteins from wheat CS and SX, respectively. Green background colour indicates sequence variance between two proteins. [file PBI-22-1989-s002.jpg]

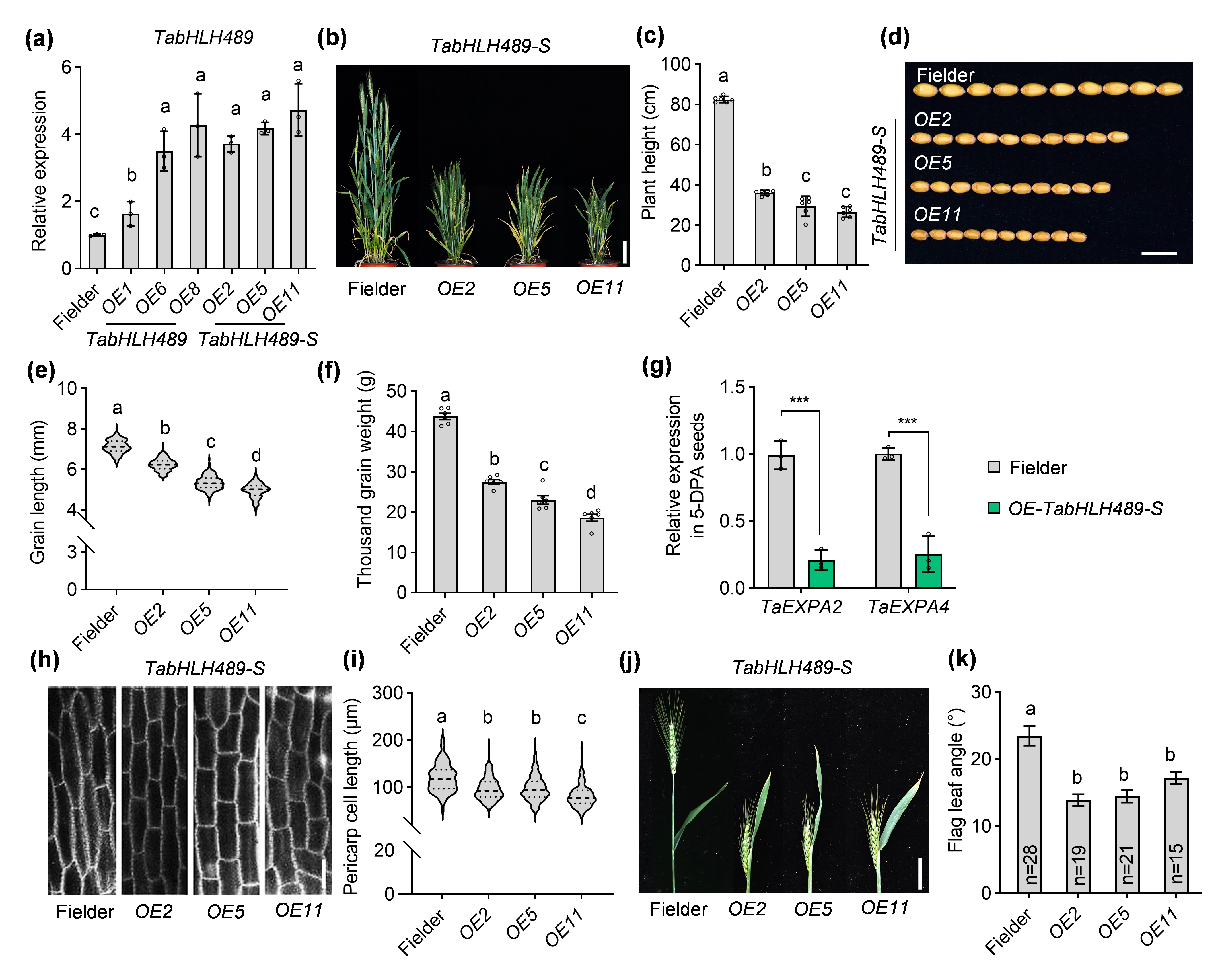

Supplement: Supplementary file 6 — Figure S6 The overexpression of TabHLH489 from SX resulted in the decreased grain length and grain weight. (a) Quantitative RT‐PCR analysis of the expression level of TabHLH489 in 5‐DPA seeds of OE‐TabHLH489 and OE‐TabHLH489‐S overexpression plants. Error bars indicate ±SD from three biological repeats. TaADPRF was used as an internal control. (b, c) Plant architecture of Fielder and OE‐TabHLH489‐S plants at the heading stage. Error bars indicate ±SD (n ≥ 6). Scale bar = 10 cm. (d–f) Wheat grain morphology of Fielder and OE‐TabHLH489‐S lines. The grains (n > 100) came from six individual plants on average. Scale bar = 1 cm. (g) Quantitative RT‐PCR analysis of TaEXPA2 and TaEXPA4 in 5‐DPA seeds of Fielder, OE‐TabHLH489‐S plants. Error bars indicate ±SD (n = 3). TaADPRF was used as an internal control. (h, i) The grain pericarp cell length of Fielder and OE‐TabHLH489‐S lines. The cells (n > 100) came from six individual plants on average. Scale bar = 50 μm. (j, k) The flag leaf angles of Fielder and OE‐TabHLH489‐S lines. Error bars indicate ±SE, Scale bar = 5 cm. Different letters above bars indicate statistically significant differences between samples (one‐way ANOVA, P < 0.05). ‘***’ indicates statistically significant differences between samples (Student's t‐test, P < 0.001). [file PBI-22-1989-s012.jpg]

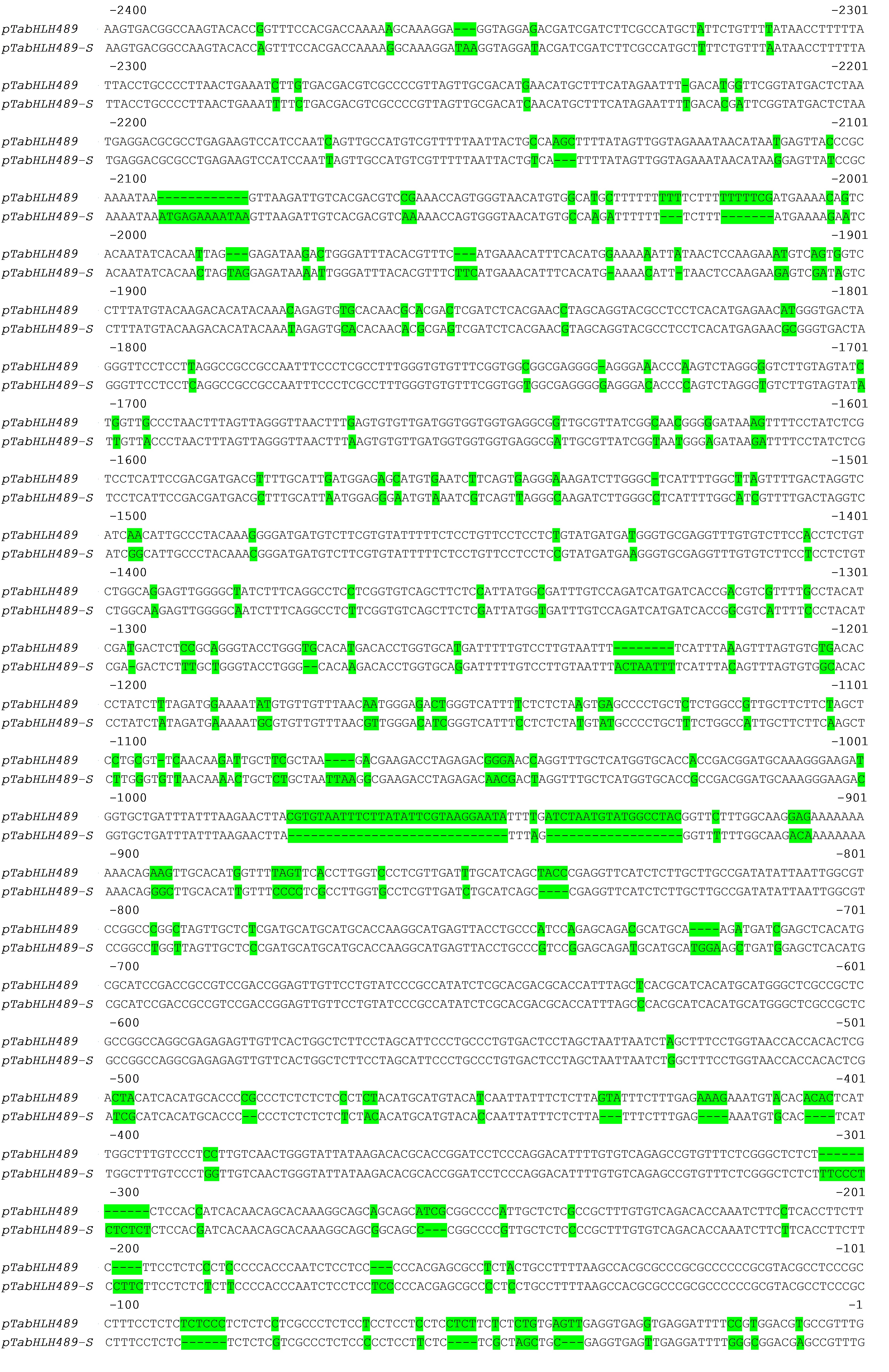

Supplement: Supplementary file 7 — Figure S7 Sequence variations between the promoters of TabHLH489 from CS and SX. pTabHLH489 and pTabHLH489‐S indicate promoters from wheat CS and SX, respectively. Green background colour indicates the sequence variance between two promoters. [file PBI-22-1989-s011.jpg]

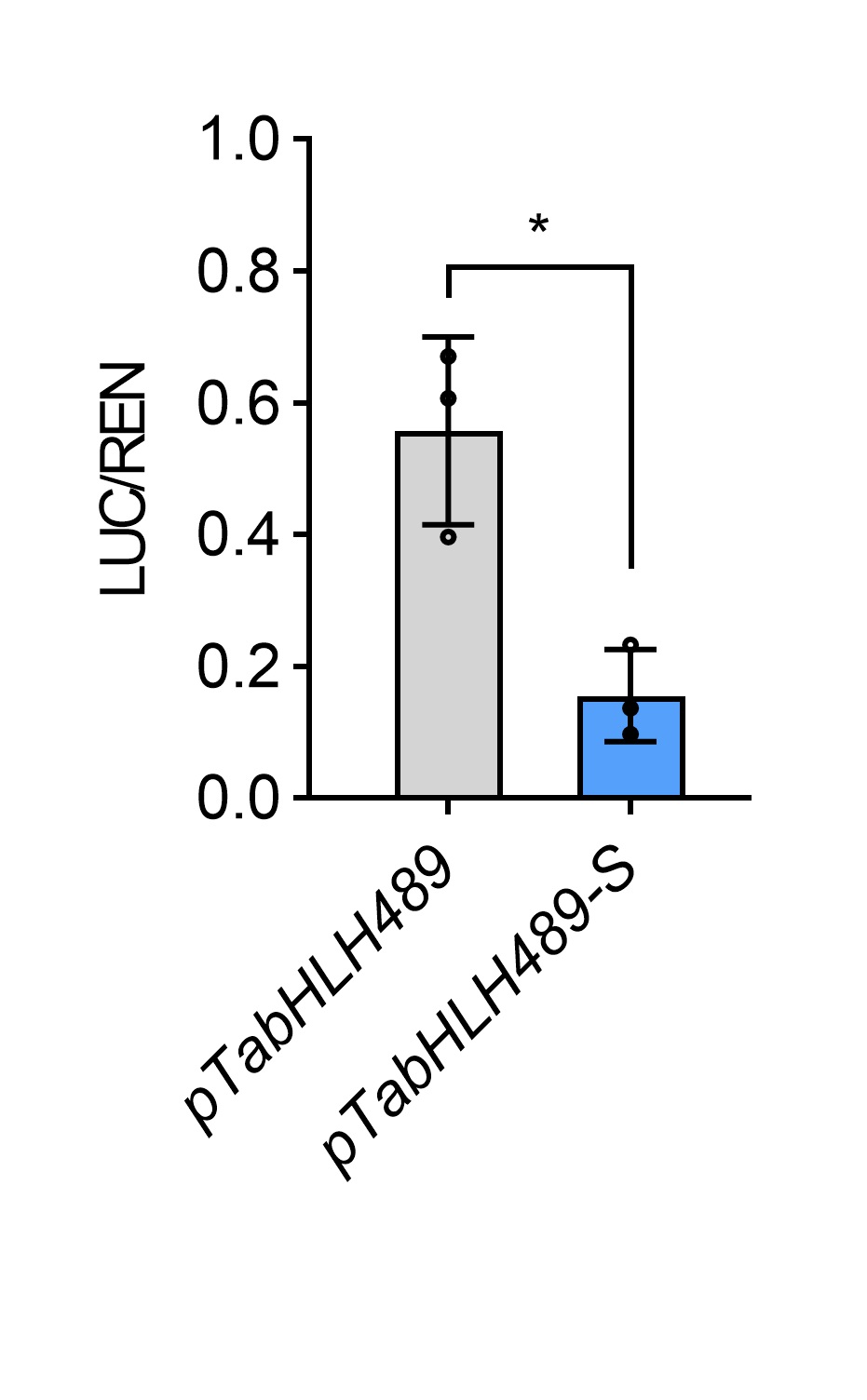

Supplement: Supplementary file 8 — Figure S8 The promoter activity of pTabHLH489 and pTabHLH489‐S. LUC activity was normalized to REN. Error bars indicate ±SD (n = 3). ‘*’ indicate statistically significant differences between two samples (Student's t‐test, P < 0.05). [file PBI-22-1989-s014.jpg]

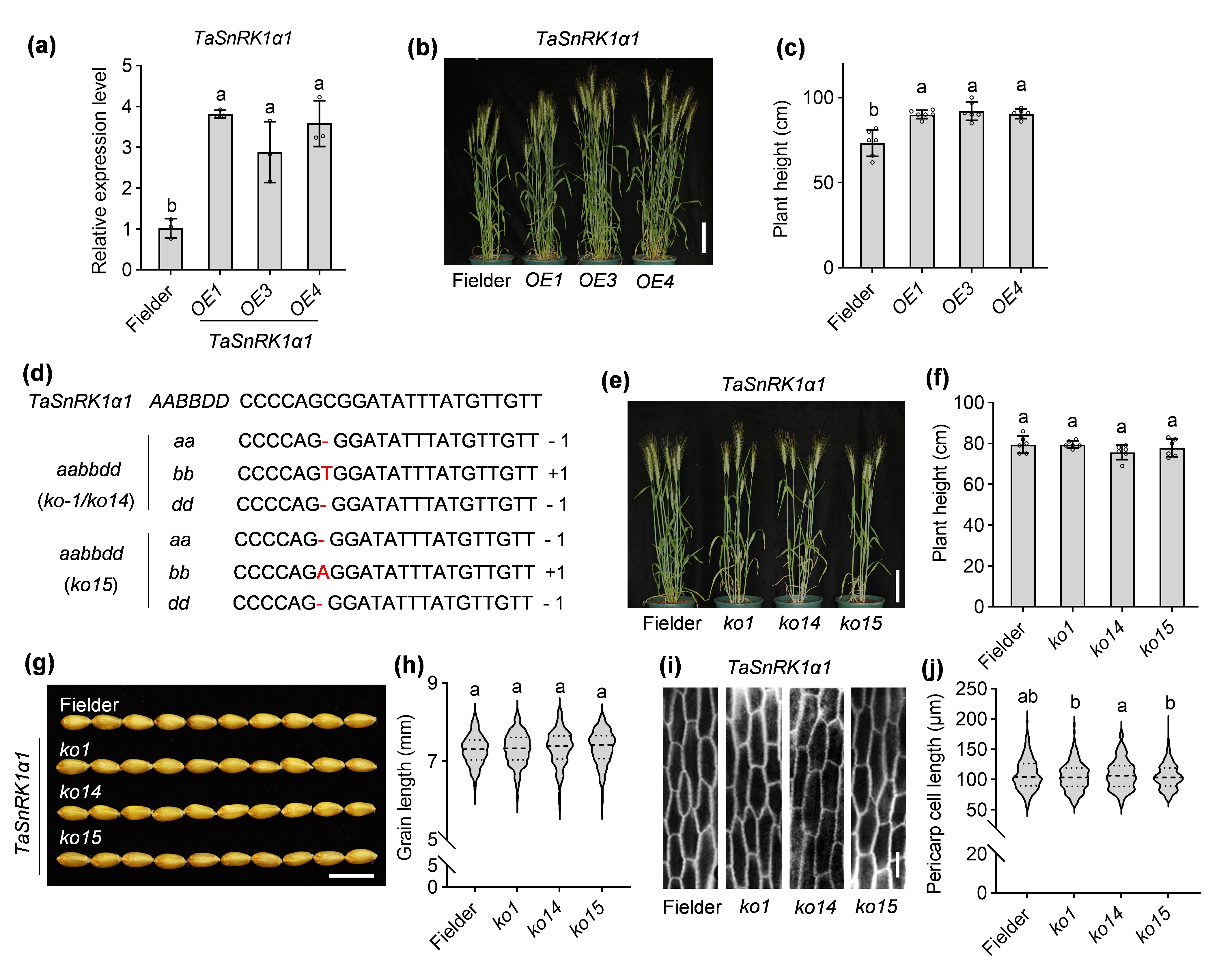

Supplement: Supplementary file 9 — Figure S9 TaSnRK1α1 promotes wheat grain development. (a) Quantitative RT‐PCR analysis of the expression level of TaSnRK1α1 in 5‐DPA seeds of Fielder and OE‐TaSnRK1α1 plants. Error bars indicate ±SD from three biological repeats. TaADPRF was used as an internal control. (b, c) Plant architecture of Fielder and OE‐TaSnRK1α1 plants at the heading stage. Error bars indicate ±SD (n ≥ 6). Scale bar = 10 cm. (d) The mutation sites of tasnrk1α1‐ko mutants are indicated in red. (e, f) Plant architecture of Fielder and tasnrk1α1‐ko mutants at the heading stage. Error bars indicate ±SD (n ≥ 6). Scale bar = 10 cm. (g, h) Wheat grain morphology of Fielder and tasnrk1α1‐ko mutants. The grains (n > 300) came from six individual plants on average. Scale bar = 1 cm. (i, j) The grain pericarp cell length of Fielder and tasnrk1α1‐ko mutants. The cells (n > 100) came from six individual plants on average. Scale bar = 50 μm. Different letters above bars indicate statistically significant differences between samples (one‐way ANOVA, P < 0.05). [file PBI-22-1989-s015.jpg]

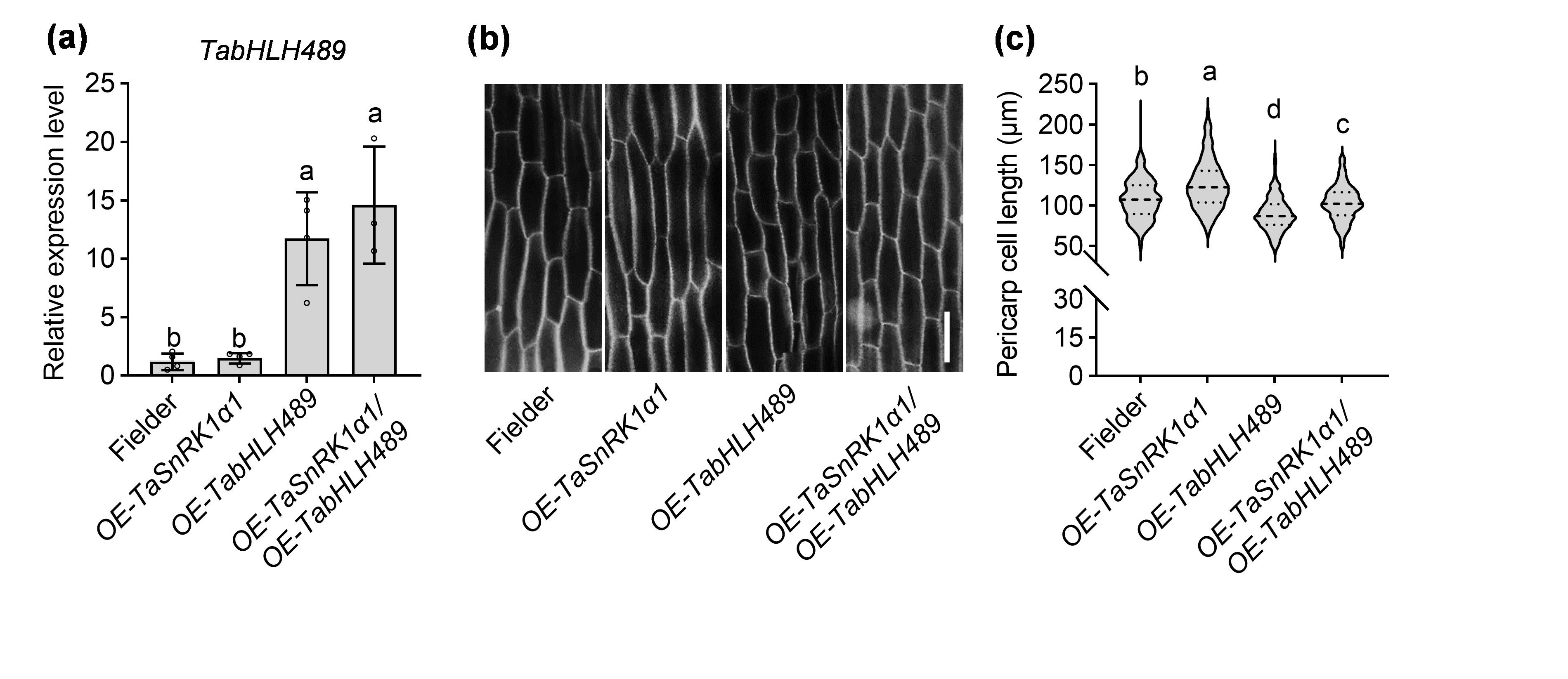

Supplement: Supplementary file 10 — Figure S10 TaSnRK1α1 inhibits activity of TabHLH489 in the crossing progenies. (a) Quantitative RT‐PCR analysis of the expression level of TabHLH489 in 5‐DPA seeds of Fielder, OE‐TaSnRK1α1, OE‐TabHLH489 and OE‐TaSnRK1α1/OE‐TabHLH489 plants, respectively. Error bars indicate ±SD from three biological repeats. TaADPRF was used as an internal control. (b, c) The grain pericarp cell length of Fielder, OE‐TabHLH489, OE‐TaSnRK1α1 and OE‐TaSnRK1α1/OE‐TabHLH489 plants. The cells (n > 100) came from six individual plants on average. Scale bar = 50 μm. Different letters above bars indicate statistically significant differences between samples (one‐way ANOVA, P < 0.05). [file PBI-22-1989-s005.jpg]

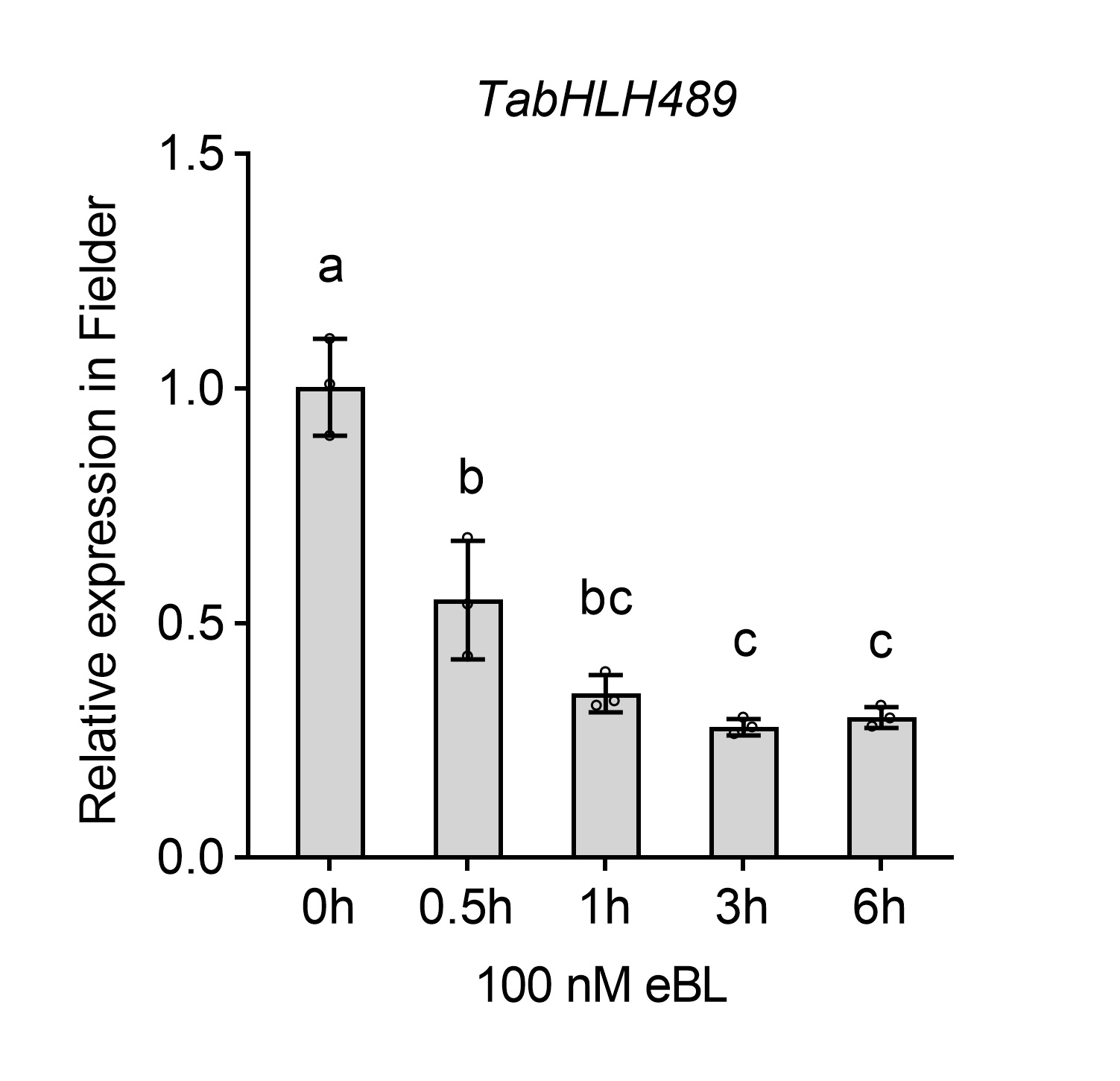

Supplement: Supplementary file 11 — Figure S11 Quantitative RT‐PCR analysis of BR effects on TabHLH489 expression. Total RNA was extracted from the leaves of 7‐day‐old seedlings which were foliar‐sprayed with 100 nM eBL (containing 0.01% TritonX‐100) for different time. Error bars indicate ±SD (n = 3). TaADPRF was used as an internal control. Different letters above bars indicate statistically significant differences between samples (one‐way ANOVA, P < 0.05). [file PBI-22-1989-s019.jpg]

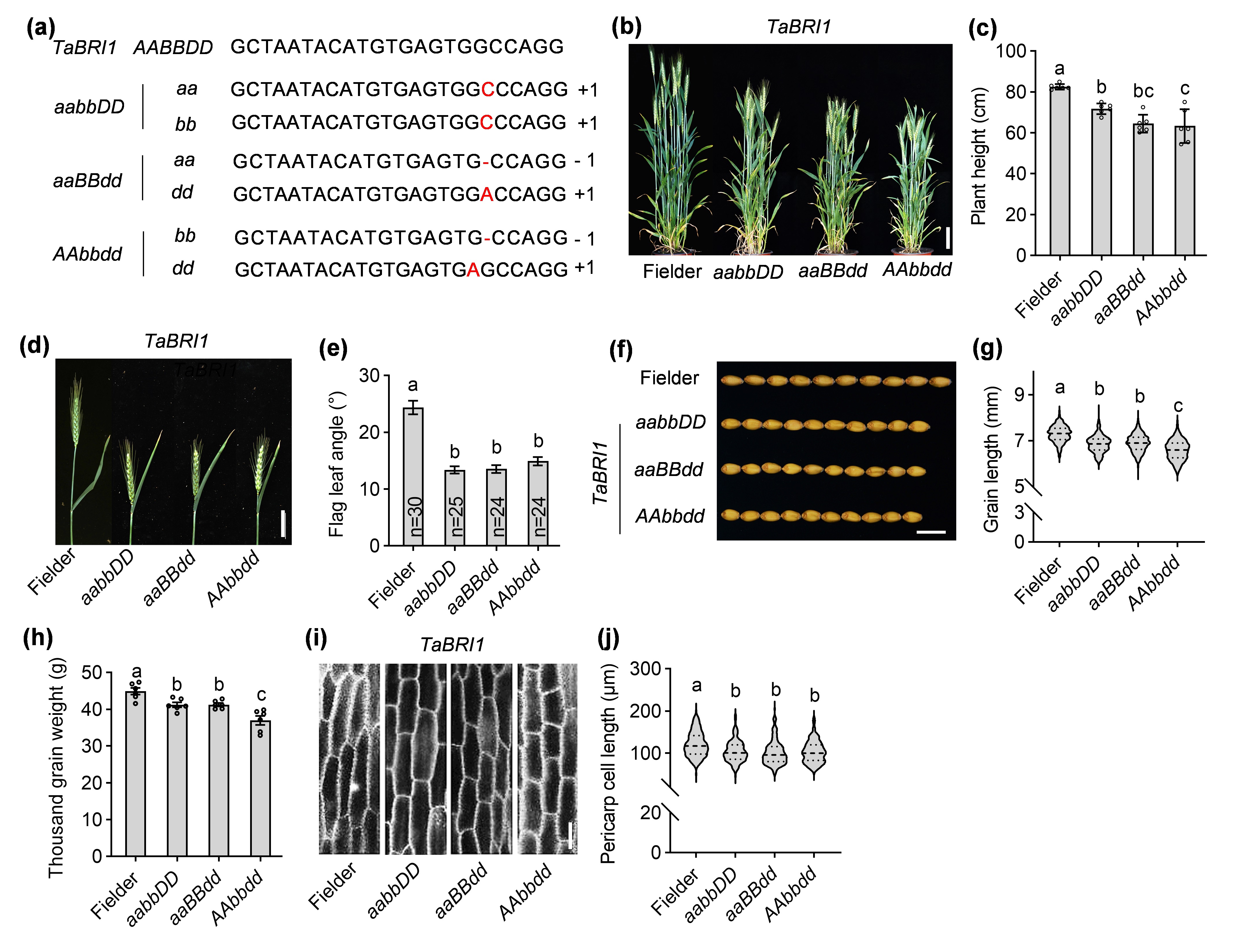

Supplement: Supplementary file 12 — Figure S12 TaBRI1 knocked out affects wheat grain development. (a) The mutation sites of TaBRI1 knockout mutants are indicated in red. (b, c) Plant architecture of Fielder and tabri1 mutants at the heading stage. Error bars indicate ±SD (n ≥ 6). Scale bar = 10 cm. (d, e) The flag leaf angles of Fielder and tabri1 mutants. Scale bar = 5 cm. Error bars indicate ±SE of different lines. (f–h) Wheat grain morphology of Fielder and tabri1 mutants. The grains (n > 300) came from six individual plants on average. Scale bar = 1 cm. (i, j) The grain pericarp cell length of Fielder and tabri1 mutants. The cells (n > 100) came from six individual plants on average. Scale bar = 50 μm. Different letters above bars indicate statistically significant differences between samples (one‐way ANOVA, P < 0.05). [file PBI-22-1989-s018.jpg]

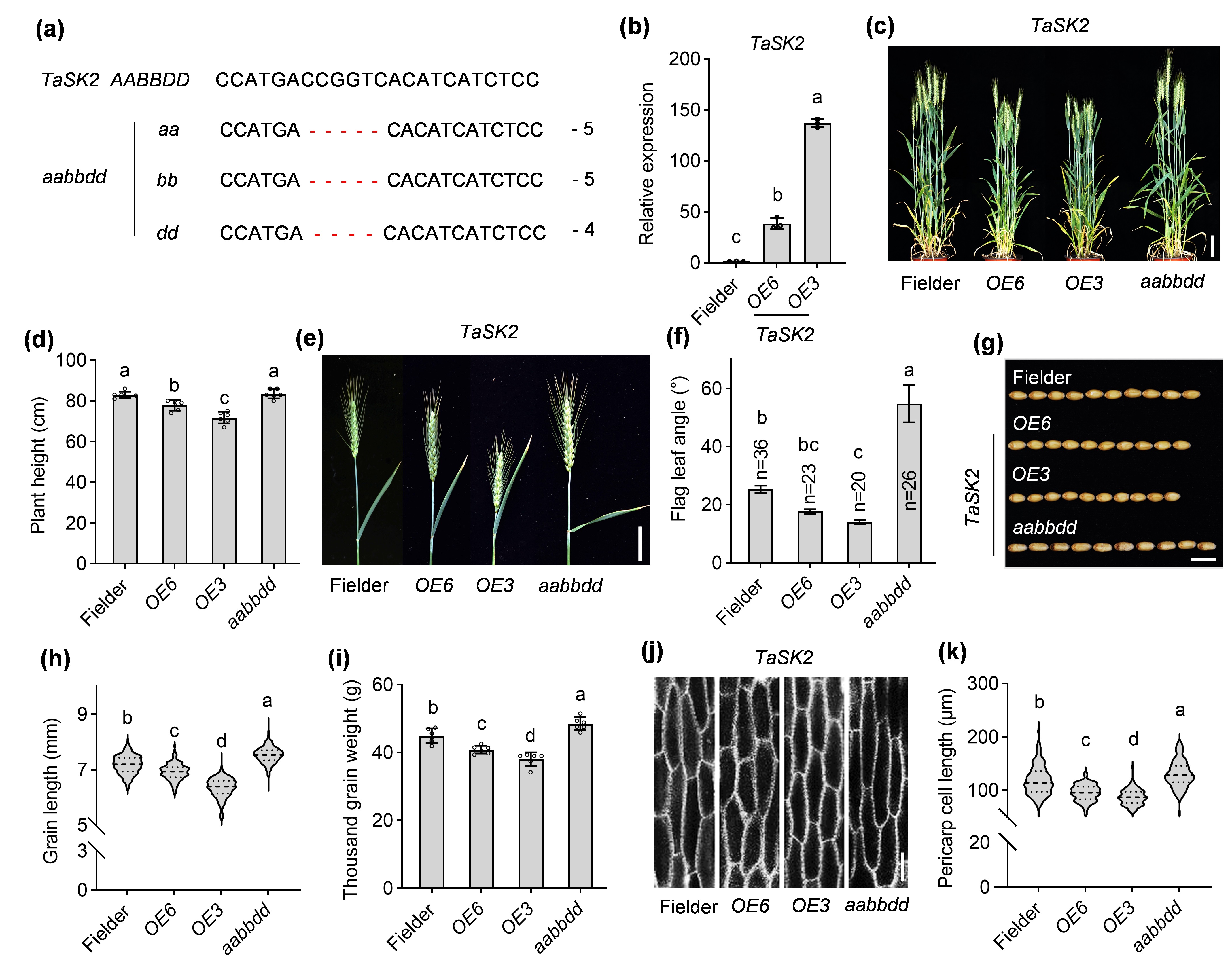

Supplement: Supplementary file 13 — Figure S13 TaSK2 inhibited wheat grain development. (a) The mutation sites of TaSK2 mutant are indicated in red. (b) Quantitative RT‐PCR analysis of the expression level of TaSK2 in 5‐DPA seeds of OE‐TaSK2 plants. Error bars indicate ±SD from three biological repeats. TaADPRF was used as an internal control. (c, d) Plant architecture of Fielder, OE‐TaSK2 and task2‐aabbdd mutant at the heading stage. Error bars indicate ±SD (n ≥ 6). Scale bar = 10 cm. (e, f) The flag leaf angles of Fielder, OE‐TaSK2 and task2‐aabbdd mutant. Scale bar = 5 cm. Error bars indicate ±SE. (g–i) Wheat grain morphology of Fielder, OE‐TaSK2 and task2‐aabbdd mutant. The grains (n > 300) came from six individual plants on average. Scale bar = 1 cm. (j, k) The grain pericarp cell length of Fielder, OE‐TaSK2 and task2‐aabbdd mutant. The cells (n > 100) came from six individual plants on average. Scale bar = 50 μm. Different letters above bars indicate statistically significant differences between samples (one‐way ANOVA, P < 0.05). [file PBI-22-1989-s008.jpg]

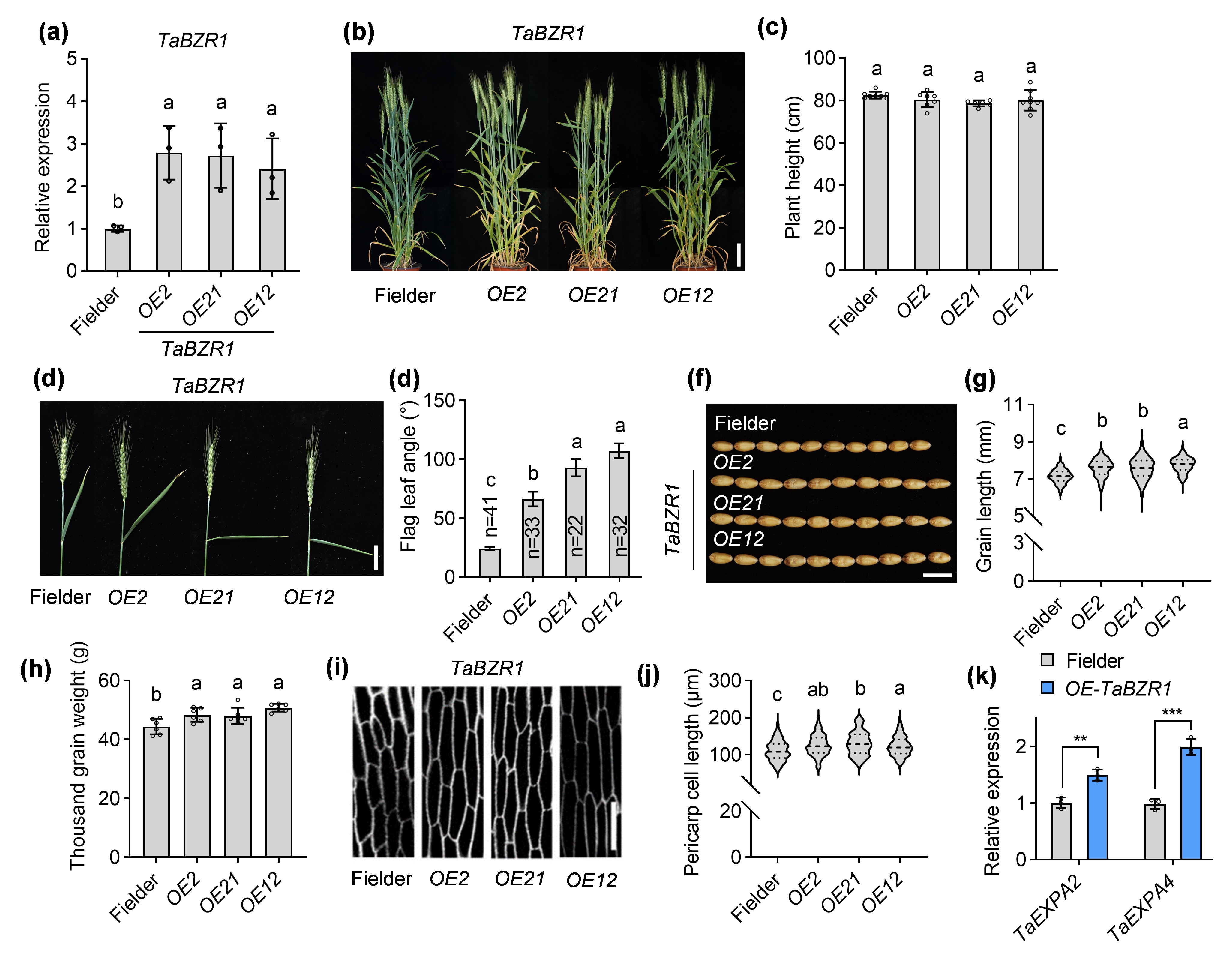

Supplement: Supplementary file 14 — Figure S14 TaBZR1 promotes wheat grain development. (a) Quantitative RT‐PCR analysis of the expression level of TaBZR1 in 5‐DPA seeds of Fielder and OE‐TaBZR1 plants. Error bars indicate ±SD from three biological repeats. TaADPRF was used as an internal control. (b, c) Plant architecture of Fielder and OE‐TaBZR1 plants at the heading stage. Error bars indicate ±SD (n ≥ 6). Scale bar = 10 cm. (d, e) The flag leaf angles of Fielder and OE‐TaBZR1 plants. Error bars indicate ±SE, Scale bar = 5 cm. (f–h) Wheat grain morphology of Fielder and OE‐TaBZR1 plants. The grains (n > 300) came from six individual plants on average. Scale bar = 1 cm. (i, j) The grain pericarp cell length of Fielder and OE‐TaBZR1 plants. The cells (n > 100) came from six individual plants on average. Scale bar = 100 μm. (k) Quantitative RT‐PCR analysis of TaEXPA2 and TaEXPA4 in 5‐DPA seeds of Fielder and OE‐TaBZR1 plants. Error bars indicate ±SD (n = 3). TaADPRF was used as an internal control. Different letters above bars indicate statistically significant differences between samples (one‐way ANOVA, P < 0.05). ‘**’ and ‘***’ indicates statistically significant differences between samples (Student's t‐test, P < 0.01 and P < 0.01, respectively). [file PBI-22-1989-s010.jpg]

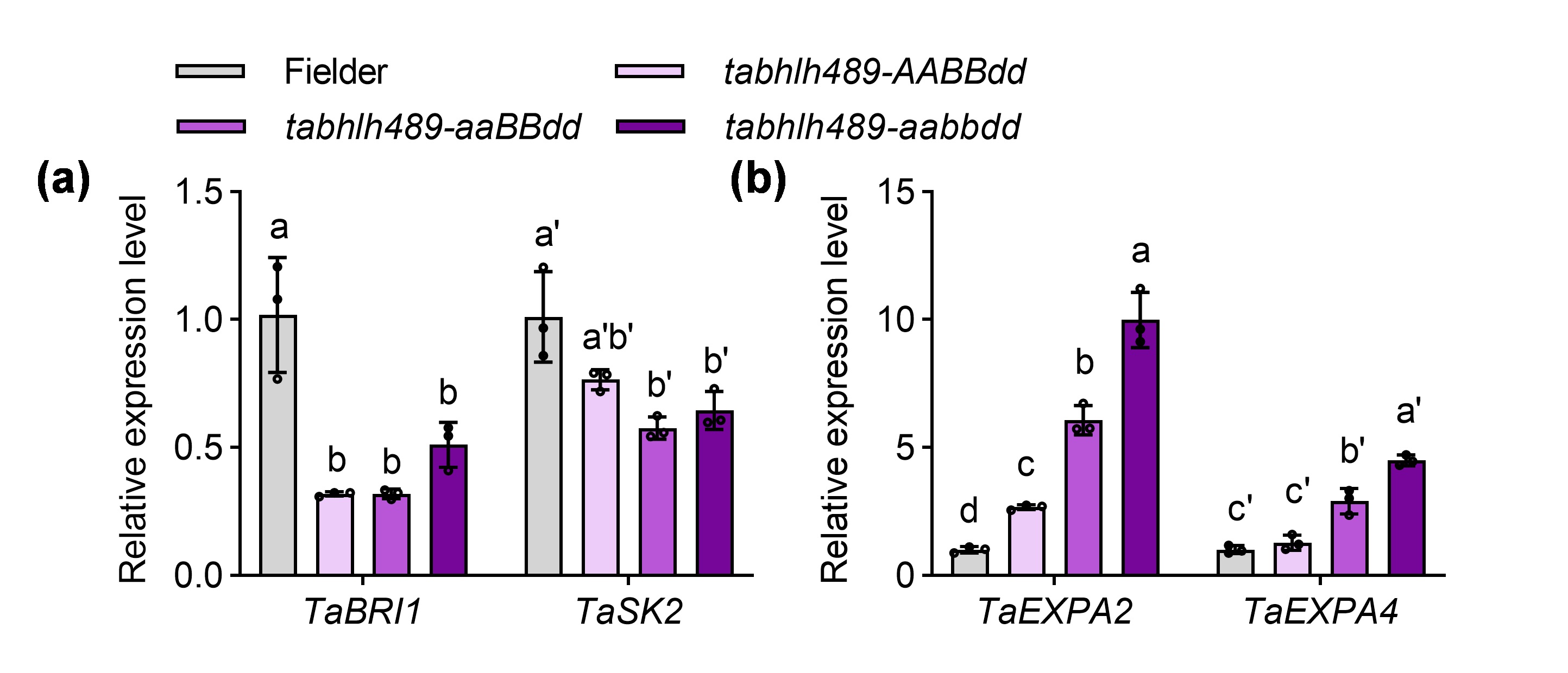

Supplement: Supplementary file 15 — Figure S15 TabHLH489 regulates the expression level of BR‐related genes in a dose‐dependent manner. (a) Quantitative RT‐PCR analysis of TaBRI1 and TaSK2 in 7‐day seedlings of Fielder and TabHLH489 single, double and triple mutants. (b) Quantitative RT‐PCR analysis of TaEXPA2 and TaEXPA4 in 7‐day seedlings of Fielder and TabHLH489 single, double and triple mutants. Error bars indicate ±SD (n = 3). TaADPRF was used as an internal control. Different letters above bars indicate statistically significant differences between samples (two‐way ANOVA, P < 0.05). [file PBI-22-1989-s003.jpg]

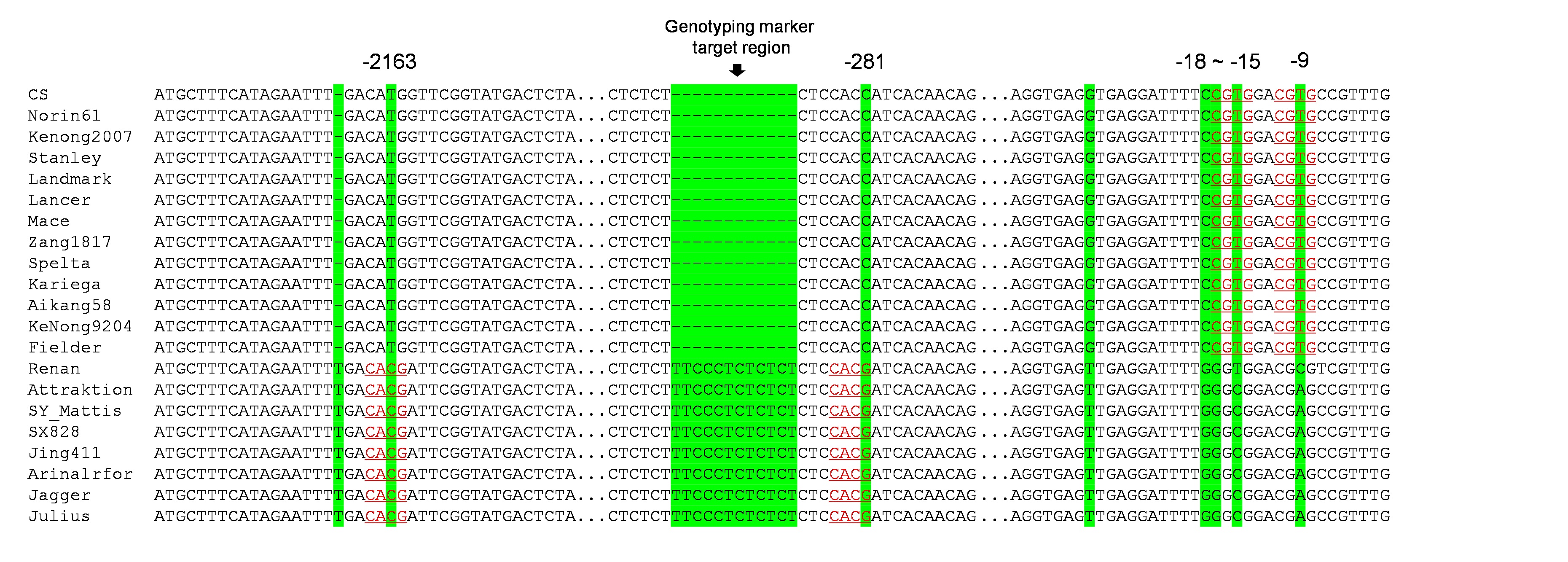

Supplement: Supplementary file 16 — Figure S16 Natural variations of the TabHLH489 promoter region alter the binding site of TaBZR1. Green background colour indicates sequence variance. Red Letters indicate the core element of the binding site of TaBZR1. [file PBI-22-1989-s017.jpg]

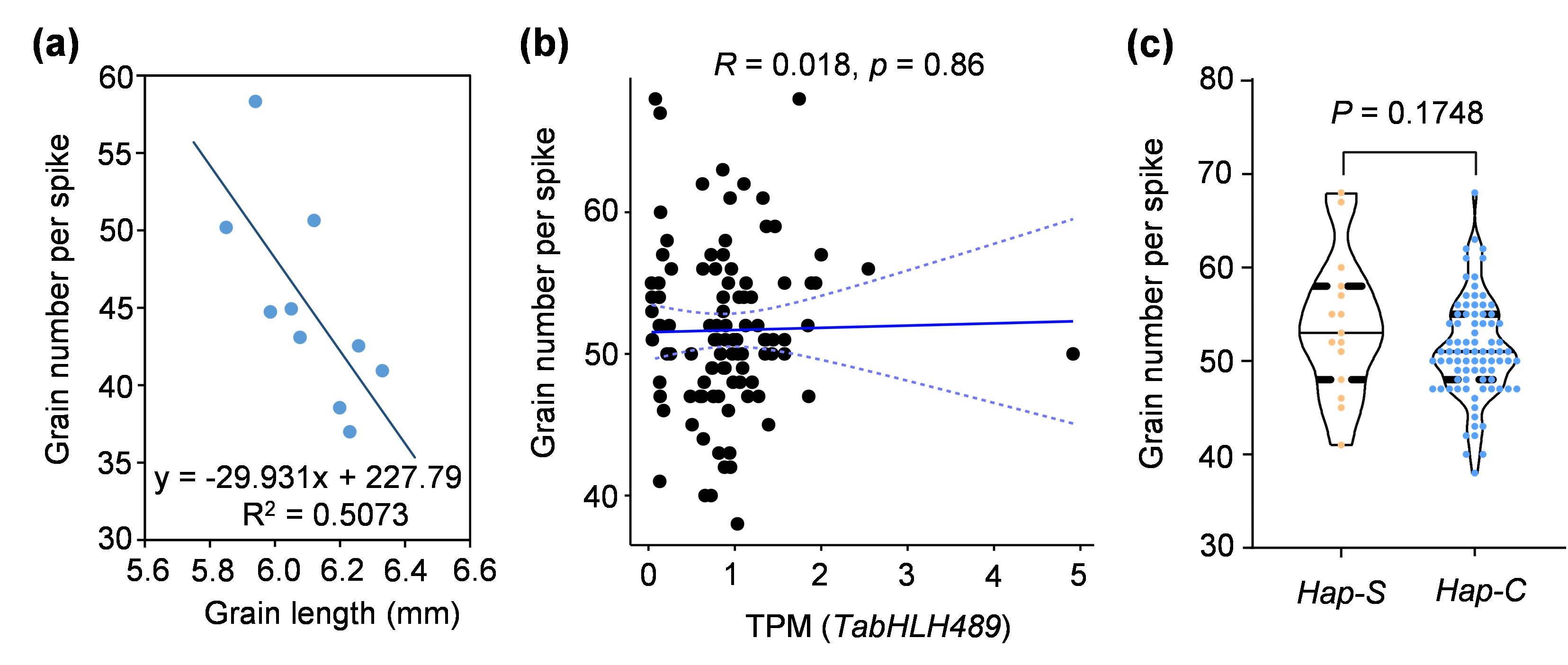

Supplement: Supplementary file 17 — Figure S17 TabHLH489 contributes a limitation of the trade‐off between grain number per spike and 1000‐grain weight. (a) The correlation analysis between the grain length and grain number per spike in wheat varieties from the major Chinese agro‐ecological zones. (b) The correlation analysis between the expression level of TabHLH489 and grain number per spike in 102 representative varieties. (c) Grain number per spike of wheat varieties containing Hap‐S or Hap‐C. [file PBI-22-1989-s006.jpg]
